# Gene repression via multiplex gRNA strategy in *Y. lipolytica*

**DOI:** 10.1186/s12934-018-0909-8

**Published:** 2018-04-20

**Authors:** Jin-lai Zhang, Yang-Zi Peng, Duo Liu, Hong Liu, Ying-Xiu Cao, Bing-Zhi Li, Chun Li, Ying-Jin Yuan

**Affiliations:** 1 Key Laboratory of Systems Bioengineering (Ministry of Education), Tianjin University, Tianjin, 300072 People’s Republic of China; 2 SynBio Research Platform, Collaborative Innovation Center of Chemical Science and Engineering (Tianjin), School of Chemical Engineering and Technology, Tianjin University, Tianjin, 300072 People’s Republic of China

**Keywords:** CRISPR interference, Multiplex gene repression, *Yarrowia lipolytica*, Synthetic biology

## Abstract

**Background:**

The oleaginous yeast *Yarrowia lipolytica* is a promising microbial cell factory due to their biochemical characteristics and native capacity to accumulate lipid-based chemicals. To create heterogenous biosynthesis pathway and manipulate metabolic flux in *Y. lipolytica*, numerous studies have been done for developing synthetic biology tools for gene regulation. CRISPR interference (CRISPRi), as an emerging technology, has been applied for specifically repressing genes of interest.

**Results:**

In this study, we established CRISPRi systems in *Y. lipolytica* based on four different repressors, that was DNase-deactivated Cpf1 (dCpf1) from *Francisella novicida*, deactivated Cas9 (dCas9) from *Streptococcus pyogenes*, and two fusion proteins (dCpf1-KRAB and dCas9-KRAB). Ten gRNAs that bound to different regions of *gfp* gene were designed and the results indicated that there was no clear correlation between the repression efficiency and targeting sites no matter which repressor protein was used. In order to rapidly yield strong gene repression, a multiplex gRNAs strategy based on one-step Golden-brick assembly technology was developed. High repression efficiency 85% (dCpf1) and 92% (dCas9) were achieved in a short time by making three different gRNAs towards *gfp* gene simultaneously, which avoided the need of screening effective gRNA *loci* in advance. Moreover, two genes interference including *gfp* and *vioE* and three genes repression including *vioA*, *vioB and vioE* in protodeoxy-violaceinic acid pathway were also realized.

**Conclusion:**

Taken together, successful CRISPRi-mediated regulation of gene expression via four different repressors dCpf1, dCas9, dCpf1-KRAB and dCas9-KRAB in *Y. lipolytica* is achieved. And we demonstrate a multiplexed gRNA targeting strategy can efficiently achieve transcriptional simultaneous repression of several targeted genes and different sites of one gene using the one-step Golden-brick assembly. This timesaving method promised to be a potent transformative tool valuable for metabolic engineering, synthetic biology, and functional genomic studies of *Y. lipolytica.*

**Electronic supplementary material:**

The online version of this article (10.1186/s12934-018-0909-8) contains supplementary material, which is available to authorized users.

## Background

Effective metabolic engineering of cell factories and technologies of genetics enables the production of biofuels and biochemical from renewable resources at low and competitive cost [1–7]. In this context, the oleaginous yeast *Yarrowia lipolytica* has become a very attractive cell factory for industrial biotechnology applications [8–19], because of its ability to natively accumulate high quantities of lipids coupled with a wide substrates portfolios [20, 21] and simple industrial scale-up operations [22]. In addition, *Y. lipolytica* is also recognized as a “generally regarded as safe” (GRAS) organism [22], which makes it a promising candidate platform for the production of high-value pharmaceutical compounds [23–26]. In order to create heterogenous biosynthesis pathway and manipulate metabolic flux in *Y. lipolytica*, a number of gene expression and deletion tools have been established in the last decade [27–33]. However, as a non-conventional yeast, the capability of selective and tunable perturbation of gene expression in *Y. lipolytica* is still rather undeveloped compared to other yeasts such as *Saccharomyces cerevisiae*, which hindered further complex design, engineering and application of this organism [34, 35].

The engineered CRISPR (clustered regularly interspaced short palindromic repeats)/Cas system has been proved its ability of highly selective transcriptional modulation over a significant dynamic range [36, 37]. Two parts are involved in the CRISPR interference (CRISPRi) system: an endonuclease-deficient, yet RNA-binding Cas protein (dCas) and a single guide RNA (gRNA). The guide sequences in gRNA is responsible for specific recognition of target gene, but the target site was determined by a PAM (Protospacer Adjacent Motif) sequence, which varied from different Cas protein [38]. Catalytically deactivated Cas9 of *Streptococcus pyogenes* (spdCas9) derived from a type II CRISPR system is the best studied and most widely used Cas protein [39–41]. The recent development of CRISPRi/dCas9 technology for *Y. lipolytica* now allows enhanced homologous recombination efficiency without labored genetic knockouts and promises to be a potent tool for other metabolic engineering [42]. However, The SpCas9 requires a G-rich PAM sequence (5-NGG-3) which is not always available in all chromosomes, more particularly in AT-rich regions [38]. Another CRISPR-Cas protein Cpf1 provides a potential solution. Cpf1 belongs to the class II type V-A CRISPR-Cas system [43–47] and recognizes a T-rich PAM at the 5′-end of the protospacer sequence [48]. Cpf1 makes a staggered double-strand break resulting in five-nucleotide 5′-overhangs distal to the PAM site [45], whereas Cas9 creates blunt ends proximal to the PAM site [49]. It is worth mentioning that the Cpf1 PAMs also vary with different sources, which describe as 5′-TTTN-3′ (or 5′-TTTV-3′) for EeCpf1 [50], AsCpf1 [51, 52], LbCpf1 [53] and 5′-TTN-3′ for FnCpf1 [45, 54]. These features propel Cpf1 as an attractive protein complementary to the Cas9 for genome editing and gene regulation [45, 54, 55]. Based on Cpf1 endonuclease mutants, CRISPRi/dCpf1 system was constructed and shown to mediate target gene repression effectively in both bacteria [50, 51] and plant cells [53].

When using CRISPRi technology to repress target genes, high-efficiency repression site was necessary. In either CRISPRi/dCas9 or CRISPRi/dCpf1 system, the repression efficiency was highly depended on the binding position of the gRNAs on the target gene. In bacteria and mammalian cell, this relationship was proved to follow certain rules. The dCas9 targeting non-template DNA strand of coding sequence with the nearest distance to transcription start site (TSS) demonstrated the most efficient gene repression in *E. coli* [36, 39, 56], *Shewanella oneidensis* MR-1 [41], cyanobacteria [57] and many other cells [37]. The dCpf1 from *Acidaminococcus* sp. and *Eubacterium eligens* also showed site-dependent rules that stronger gene repression was achieved when it was targeted to the template strand rather than non-template strand in *E. coli* [50, 51]. But in yeast, the repression efficiency of different gRNA targeting was more complicated. Onge et al. found that gRNA efficacy depended on accessibility and location of the target region, and the best region of target gRNA was between the TSS and 200 bp upstream of the TSS in *S. cerevisiae* [58]. Another CRISPRi study, however, yielded a different result that the scRNA targeting the TSS + 21 position of *erg12* achieved the most efficient repression (~ 3-fold), different from the best region Onge et al. [58] found. And from testing a total of in silico designed 88 scRNAs on 12 native yeast promoters, Jensen et al. found that high-efficiency repression site was hard to determine [59].

**Fig. 1:**
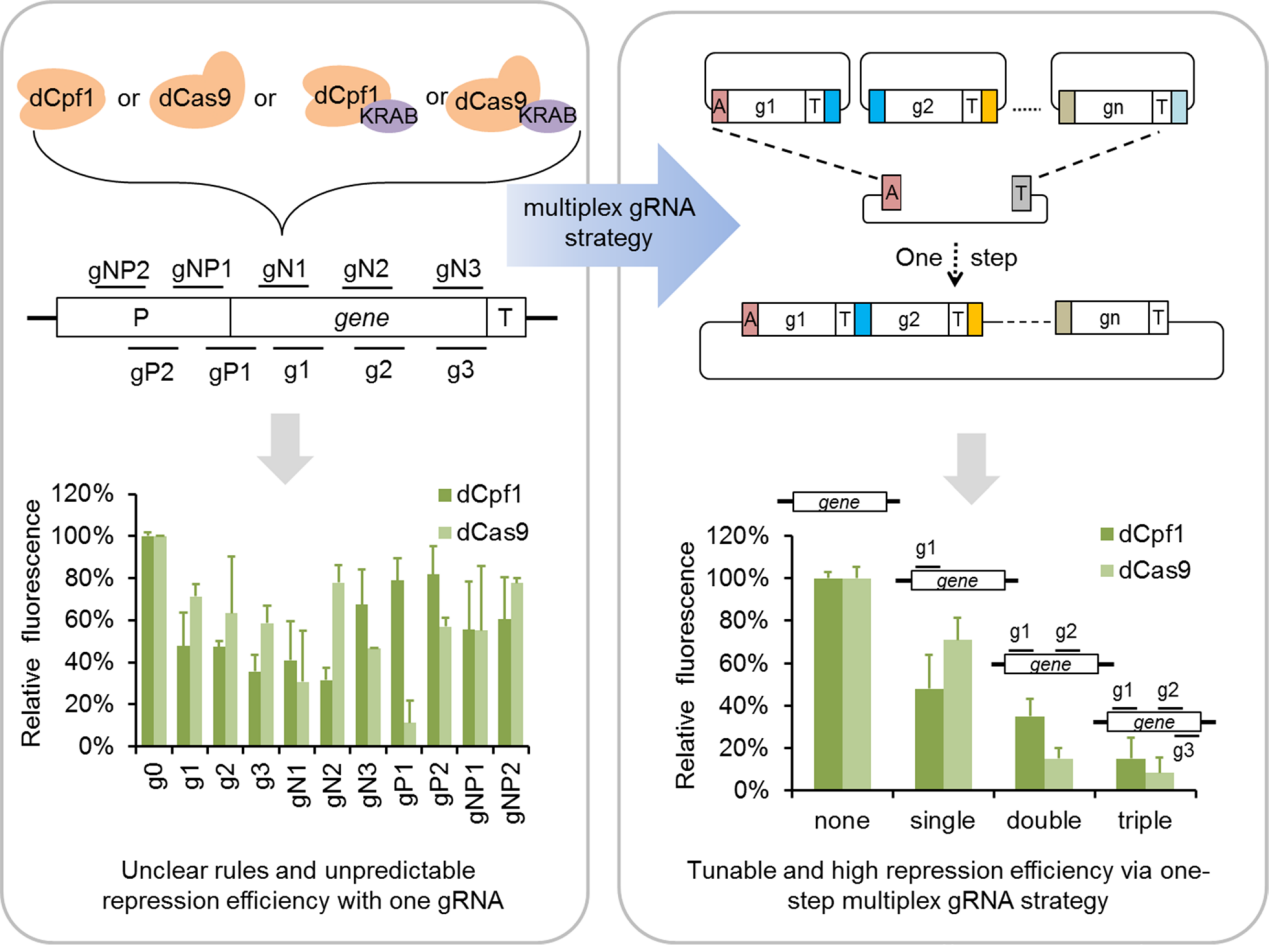
Schematic diagram of gene repression via multiplex CRISPRi system in *Y. lipolytica*. The combination of four different repressors (dCpf1, dCas9, dCpf1-KRAB and dCas9-KRAB) and ten gRNAs that bind to different regions of *gfp* gene were employed in *Y. lipolytica*. But the results indicated that there was no clear correlation between the repression efficiency and targeting sites (left). As extra target sites screening often mandated a significant investment of time and effort, a strategy via one-step Golden-brick assembly of multiplex gRNAs was established for high-efficiency and tunable perturbation of gene expression in *Y. lipolytica* (right)

In the present work, the irregular repression results related to different gRNA targeting region was also discovered in *Y. lipolytica.* We established CRISPRi systems in *Y. lipolytica* based on four different repressors. But the effects of the binding strand and location bias of the dCas-gRNA complex on the repression of gene expression were irregular in every case (Fig. 1). This phenomenon made it impossible to achieve strong or tunable gene repression in *Y. lipolytica* by simply applying one gRNA. Thus, a multiplex gRNAs strategy was exploited which was in the basis of one-step Golden-brick assembly (Fig. 1). By applying this strategy, as high as 85% (~ 6.7-fold) and 92% (~ 12.5-fold) repression efficiency were rapidly realized with dCpf1-Multi and dCas9-Multi system respectively by making three different gRNAs towards *gfp* gene simultaneously. Moreover, two genes interference including *gfp* and *vioE* and three genes repression including *vioA*, *vioB and vioE* were also realized using this method. This work developed a potent transformative tool enabling rapid inhibition of target gene expression without the need of screening a large number of target sites in advance in *Y. lipolytica*.

## Methods

### Genes, strains and culture conditions

Strains used in this study were listed in Additional file 1: Tables S1. The fragments of *dCpf1* (D917A) derived from *Francisella novicida* U112 (NC_00860 1) [45], *Cas9* and *dCas9* (D10A and H841A) derived from *Streptococcus pyogenes* [36], *dCas9*-*KRAB* [37], *dCpf1*-*KRAB*, *sfGFP* [60, 61], *VioA, VioB, VioE* [62], and vector assembled modules of pMCSCen1 [63], PMCS-Multi, JLPC/N-n and JLRC/N-n were codon optimized and synthesized by GenScript (Nanjing China). gRNA oligos were synthesized by Genewiz (Suzhou, China). *Escherichia coli* Trans1-T1 was used for plasmid construction and propagation, which was cultured in Lysogeny broth (LB) medium at 37 °C at 250 rpm. Whenever required, 100 mg/l ampicillin or 50 mg/l kanamycin was added. The ATCC 201249 MATA stain of *Y. lipolytica* was cultured in either YPD medium (1% yeast extract, 2% peptone, 2% glucose) or in synthetic complete media (SC) (0.67% yeast nitrogen base, 0.2% amino acid mixture, and 2% glucose). All *Y. lipolytica* culturing was done at 28 °C, and a shaking speed of 250 rpm was used for all liquid cultures in 25 ml polypropylene tubes or 250 ml conical flasks.

### Plasmid construction and transformation

Plasmids and primers used in this study are listed in Additional file 1: Tables S1 and Additional file 2: Table S2, respectively. All plasmids employed for gene expression in *Y. lipolytica* were centromeric replicative vectors based on plasmid pSl16-Cen1-1, which was initially modified to include a new multicloning site and redubbed pMCSCen1 [63]. The fragments of *dCpf1*, *dCas9*, *dCpf1*-*KRAB* and *dCas9*-*KRAB* were inserted into vector pMCSCen1 via restriction enzyme digestion and ligation to form corresponding plasmids (PMCS-dCpf1, PMCS-dCas9, PMCS-dCpf1-KRAB, PMCS-dCas9-KRAB).

For single repression, the synthesized gRNA was incorporated into the PMCS-dCpf1, PMCS-dCas9, PMCS-dCpf1-KRAB, PMCS-dCas9-KRAB plasmids respectively (unique BbsI restriction site) via one-step Golden Gate assembly. All plasmids had been inserted into the gRNA expression cassette, which helped realize rapid plasmid construction to target any genomic locus of interest. Detailed plasmid assembly methods were shown in Additional file 3: Data S1.

For multiple repression, the synthesized gRNA was incorporated into the JLPC/N–n or JLRC/N–n plasmids first. The resulting vectors were digested with BsaI or BsaI and NotI together to allow for synthesis as separate gBlock. The dCpf1-Multi vector or dCas9-Multi was digested with BsaI, and the resulting digestion product was mixed with the separate gBlock above in a Golden-brick Assembly reaction to yield the final dCpf1-Multi-gRNA or dCas9-Multi-gRNA plasmid.

All enzymes and enzyme substrates were purchased from New England Biolabs. Plasmid constructions were performed in *Escherichia coli* Trans1-T1. Frozen-EZ kit was used for *Y. lipolytica* transformations and transformants of *Y. lipolytica* were selected and screened for on Sc-Ura or YPD-Hph (1% yeast extract, 2% peptone, 2% glucose, 0.04% hygromycin) or Sc-Ura-Hph (0.67% yeast nitrogen base, 0.2% amino acid mixture, 2% glucose and 0.04% hygromycin) agar plates.

### Fluorescence assay

All *Y. lipolytica* strains were activated in Sc-Ura medium for 24 h, then 500 μl of each culture suspensions were transferred in 25 ml polypropylene tubes containing 5 ml fresh Sc-Ura medium for 24 h. Finally, 1 ml of each culture suspensions were transferred in 250 ml conical flask containing 50 ml fresh Sc-Ura medium. The strains were grown at 250 rpm for  24 h at 28 °C. 1 ml suspensions of each conical flasks were centrifuged at 5000 rpm for 2 min to remove the supernatant and the cells were washed and resuspended with water. Fluorescence intensity (excitation: 488 nm and emission: 530 nm) was measured using a 96-well polystyrene plates (black plate, clear bottom) (Corning Incorporated 3603, USA) after dilution into the linear range of the detector by a multi-mode microplate reader (SpectraMax M2, Molecular Devices, USA) and cell density (OD600) was measured using cuvette by UV Spectrophotometer (TU-1810) respectively.

### Pigment assay

All *Y. lipolytica* strains were activated in Sc-Ura medium for 24 h, then 500 μl of each culture suspensions were transferred in 25 ml polypropylene tubes containing 5 ml fresh Sc-Ura medium for 24 h. Finally, 1 ml of each culture suspensions were transferred in 250 ml conical flask containing 50 ml fresh Sc-Ura medium. The strains were grown at 250 rpm for 48 h at 28 °C. 3 ml suspensions of each conical flasks were centrifuged at 13,300 rpm for 10 min to remove the supernatant and the cells were washed and resuspended with 600 μl absolute ethanol. After adding glass beads (SIGMA), the mixture was shaked for 20 min, then was centrifuged at 13,300 rpm for 15 min to acquire the supernatant. Pigment intensity (absorbance: 584 nm) was measured using a 96-well polystyrene plates (black plate, clear bottom) (Corning Incorporated 3603, USA) by a multi-mode microplate reader (SpectraMax M2, Molecular Devices, USA) and cell density (OD600) was measured using cuvette by UV Spectrophotometer (TU-1810) respectively. The relationship between PVA content and relative absorbance was shown in Additional file 4: Fig. S1.

### Quantitative real-time reverse transcription polymerase chain reaction (qRT-PCR)

Total RNA was isolated from the mid-log phase cultures using the Bacterium Total RNA Extraction Kit (APEXBIO, China), according to the instruction of the manufacturer. And cDNA was synthesized using the GoScrip™ Reverse Transcription System (Promega, USA). Quantitative analyses of expression of target genes were achieved by SsoAdvanced™ SYBR^®^ Green Supermix (Bio-Rad, USA). Gene act was used for normalization. Samples were tested in triplicate using the listed primers (Additional file 5: Table S3). The data were analyzed using the 2^−ΔΔCT^ method.

## Results and discussion

### CRISPRi mediated gene repression based on four repressors in *Y. lipolytica*

To establish an effective reporter system in *Y. lipolytica*, several available fluorescent proteins were evaluated, including RedStar2, YFP [27] and GFP [60, 61]. The TEF intron (TEFin) promoter was applied to express these fluorescence genes, and the reporter functionality was determined by a multi-mode microplate reader. Of these variants, only GFP imparted remarkable fluorescence (Additional file 6: Fig. S2). Then the *gfp* reporter gene was integrated into rDNA locus forming various control strains (Additional file 7: Data S2). Six strains were selected randomly and measured by the multi-mode microplate reader as shown in Additional file 6: Fig. S2. The best performed strain (GFP-6) was selected and used as control strain called YL-GFP for expressing CRISPRi plasmids in the following works.

**Fig. 2:**
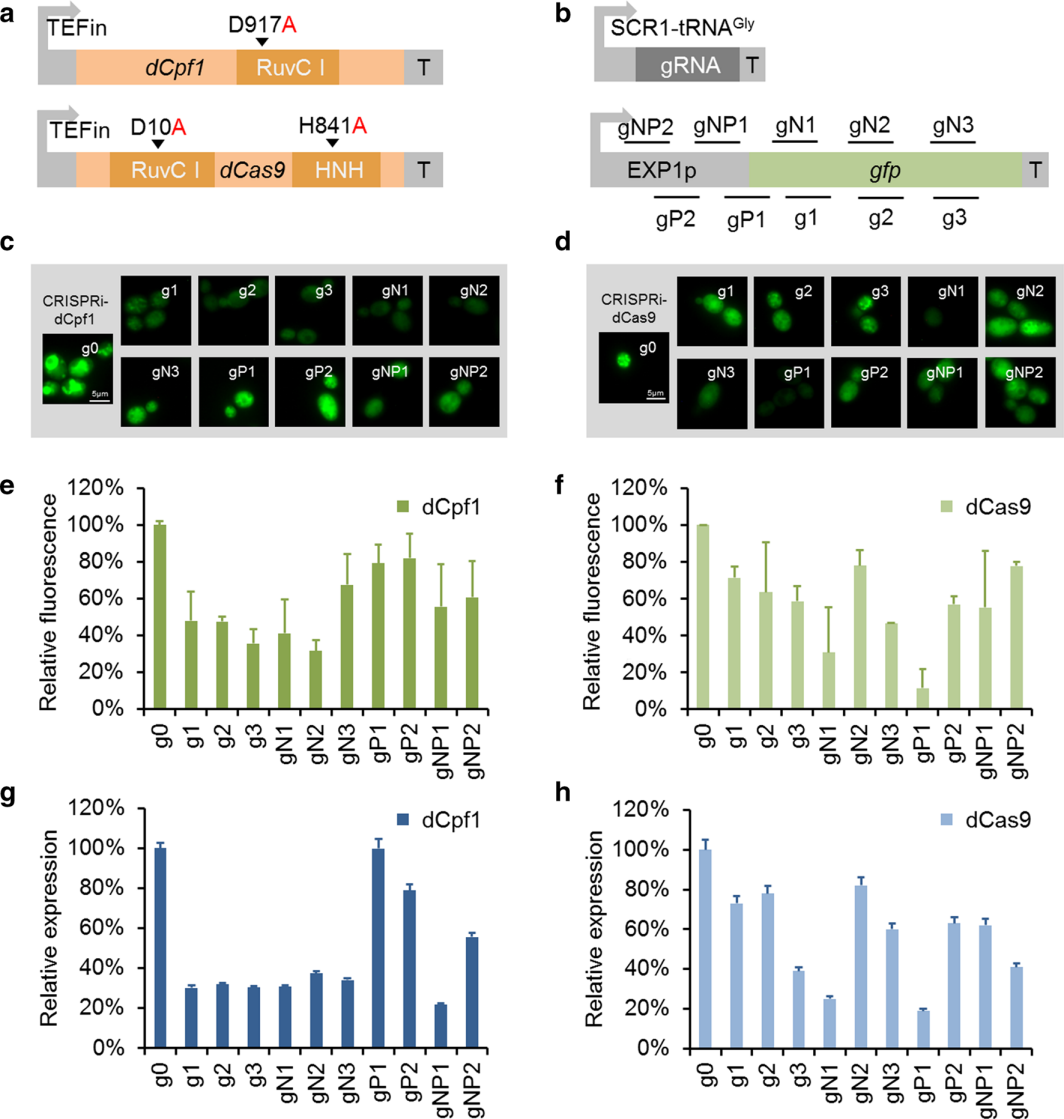
Repression of *gfp* in *Y. lipolytica* by dCpf1 and dCas9. **a** The dCpf1 and dCas9 expression system. The dCpf1 contains mutations of the RuvC1 nuclease domains while the dCas9 contains mutations of both RuvC1 and HNH nuclease domains. **b** Schematic of RNA polymerase III promoter used in this study and placement of gRNA protospacers on the target *gfp* gene. SCR1′-tRNA^Gly^ is the synthetic RNA polymerase III promoter. gRNA is single guide RNAs. polyT is a string of eight thymines, which serves as an RNA polymerase III terminator. Six gRNAs bind to either the non-template DNA strand or the template DNA strand of ORF and four gRNAs bind to different regions around the promoter. **c** Microscopic images of the interfered strains with *gfp* gene by dCpf1. **d** Microscopic images of the interfered strains with *gfp* gene by dCas9. **e** CRISPRi repression of *gfp* with dCpf1 complexed with ten gRNAs targeting different regions. The control (g0) shows fluorescence of the cells with dCpf1 protein but without the gRNA. **f** CRISPRi repression of *gfp* with dCas9 complexed with ten gRNAs targeting different regions. The control (g0) shows fluorescence of the cells with dCas9 protein but without the gRNA. **g** Characterization of the *gfp* gene’s expression level of each strain interfered by dCpf1. **h** Characterization of the *gfp* gene’s expression level of each strain interfered by dCas9. The error bars (mean ± SD) were derived from triplicate experiments for each strain

To harness the CRISPRi system for gene regulation in *Y. lipolytica*, two PMCS-CRI expression plasmids were constructed which were derived from a precursor pMCSCen1 plasmid [63] harboring cen1-1 origin and URA3 marker: The pMCS-dCpf1 plasmid included a gRNA expression cassette and *dCpf1* gene, encoding a nuclease-deficient form of the Cpf1 from *Francisella novicida* U112 (NC_00860 1) [45]. And pMCS-dCas9 plasmid was designed by utilizing a nuclease-deficient Cas9 (dCas9) from *Streptococcus pyogenes* [36] coupled with a gRNA secretion cassette. In both plasmids, *dCpf1* or *dCas9* was codon-optimized for *Y. lipolytica* and controlled by a strong, endogenous TEFin promoter (Fig. 2a), and gRNA was transcribed with synthetic hybrid Pol III promoter SCR1′-tRNA^Gly^ [31, 42] (Fig. 2b). To facilitate seamless, one-step Golden Gate assembly of custom-designed spacers (unique BbsI restriction site) were inserted into the gRNA expression cassette of both plasmids allowing rapid plasmid construction to target any genomic locus of interest. The GFP-based reporter system created above was used to testify whether the CRISPRi system could make an efficient gene repression in *Y. lipolytica*. We designed ten gRNAs complementary to different regions of the *gfp* coding sequence and promoter, either binding to the template DNA strand or to the non-template DNA strand (Fig. 2b). The microscopic images of the interfered strains by dCpf1 were shown in Fig. 2c and fluorescence result was presented in Fig. 2e. As shown, fluorescence of all strains decreased no matter gRNAs bound to coding sequence or promoter region, but different targeting sites led to different repression efficiency, ranging from below 18% (gP2) to 68% (gN2). The mRNA expression levels of influenced *gfp* gene were quantified by RT-qPCR, and the transcriptional level was consistent to the fluorescence result with the highest repression efficiency of 78% (Fig. 2g). Overall, these results indicated that dCpf1-CRISPRi can be employed as an effective tool for gene expression regulation in *Y. lipolytica*. However, the relationship between the repression efficiency and the location of targeting site was not as regular as that in bacteria [50, 51] or plant cells [53] did. As shown in Fig. 2e, different interfered *Y. lipolytica* cells showed promiscuous fluorescence results no matter gRNAs targeted template DNA strand or non-template DNA strand. Moreover, the distance of gRNA binding site to transcription start site showed no obvious impact on gene repression. To testify whether this unique phenomenon was caused by dCpf1 protein, the dCas9 protein was applied for the GFP-mediated system. We compared it with dCpf1-CRISPRi system by making dCas9 gRNAs target some positions including coding sequence or promoter region as the dCpf1-CRISPRi system did. And Fig. 2d showed the microscopic images of the interfered strains by dCas9 which demonstrated the repression functionality of dCas9 protein. The corresponding fluorescence result was shown in Fig. 2f and the mRNA expression levels of *gfp* gene were quantified by RT-qPCR (Fig. 2h). dCas9 reduced the expression of the GFP up to 89% (gP1), a more pronounced repression than that observed for other targeting, but still in low efficiency and no regular rules shown between repression efficiency and targeting sites.

**Fig. 3:**
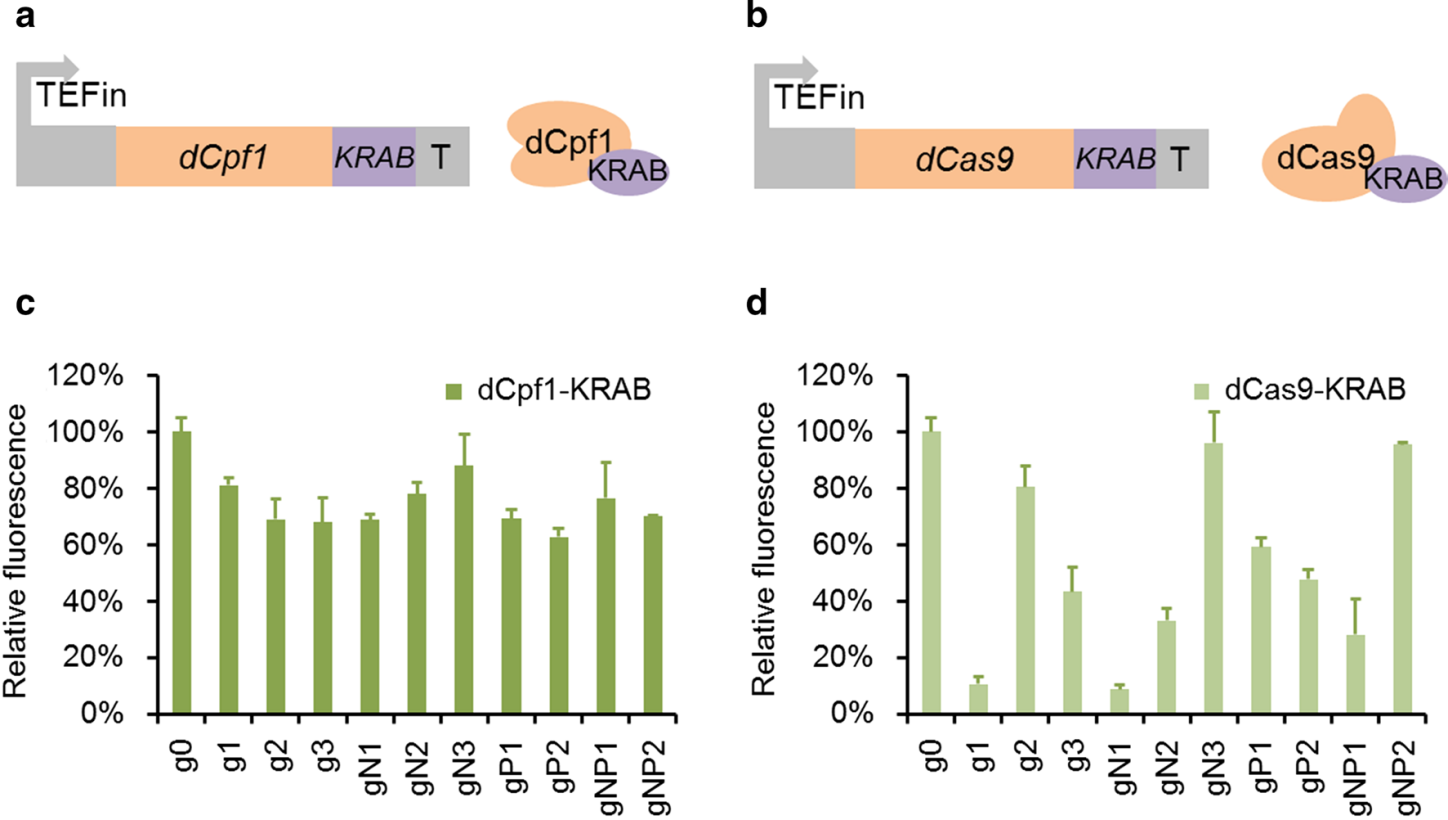
Repression of *gfp* in *Y. lipolytica* by dCpf1-KRAB and dCas9-KRAB. **a** Schematic of dCpf1 fused to repressor domain KRAB and the dCpf1-KRAB expression system. **b** Schematic of dCas9 fused to repressor domain KRAB and the dCas9-KRAB expression system. **c** CRISPRi repression of *gfp* with dCpf1-KRAB complexed with ten gRNAs targeting different regions. The control (g0) shows fluorescence of the cells with dCpf1-KRAB protein but without the gRNA. **d** CRISPRi repression of *gfp* with dCas9-KRAB complexed with ten gRNAs targeting different regions. The control (g0) shows fluorescence of the cells with dCas9-KRAB protein but without the gRNA. The error bars (mean ± SD) were derived from triplicate experiments for each strain

To yield stronger gene repression in *Y. lipolytica*, the Krüppel associated box (KRAB) domain [64] was fused to the C-terminus of dCpf1 and dCas9 respectively (Fig. 3a, b). We compared them with dCpf1-CRISPRi and dCas9-CRISPRi system by making gRNAs target the same positions including coding sequence and promoter region. The corresponding fluorescence results of dCpf1-KRAB and dCa9-KRAB were presented in Fig. 3c, d respectively. As shown, the highest repression efficiency was 91% when dCas9-KRAB was applied, which did not show much improvement compared with dCas9 (89%). Whereas the fluorescence of interfered strains by dCpf1-KRAB unexpectedly increased as a whole compared with dCpf1 and the most repression efficiency (37%) was pretty lower than it (68%) in dCpf1-CRISPRi system. However, no matter the repression efficiency increased or decreased, it could be clearly seen that the most efficient repression site varied irregularly as gN2, gP1, gP2, gN1 and g1 in the dCpf1, dCas9, dCpf1-KRAB, dCas9-KRAB and dCas9-Mxi1 system (Additional file 8: Fig. S3). These results demonstrated that there was no clear correlation between the repression efficiency and targeting sites no matter which repressor protein was used. Therefore, the highest inhibition site was hardly determined without screening gRNA *loci* in advance. Meanwhile, the repression of the endogenous gene (*pex10*) in *Y. lipolytica* was also realized and the mRNA expression levels of *pex10* gene of the interfered strains were quantified by RT-qPCR (Additional file 9: Fig. S4). The result showed the repression efficiency of *pex10* gene varied among different region but had no regular relation with the location of targeting site like the situation of *gfp* repression. As for why this happened, we speculated that one reason is the uncertain binding ability of Cas/gRNA complex on the gene loci of genome. Therefore, we constructed Cas9-pex10 plasmids for *pex10* gene knockout (Additional file 10: Data S3) with the same gRNAs in dCas9 situation to compare the disruption ratio and repression ratio. The result was presented in Additional file 9: Fig. S4 and showed that the binding ability of Cas9/gRNA complex on the gene loci of genome varied in an irregular pattern. The other reason for this phenomenon we speculated was the unique gRNA expression cassette used in CRISPRi system. *Y. lipolytica* required hybrid Pol III promoter combined with tRNA to release gRNA, forming a two-phase mature mechanism of gRNA, thus resulting the coexistence and interaction of tRNA-gRNA and gRNA [31]. This kind of coexistence and interaction made the valid integration of gRNA and dCas protein unpredictable, which consequently leading to the irregular repression results among different targeting sites.

### Enhanced *gfp* repression by one-step Golden-brick assembly of multiplex gRNAs

When using CRISPRi technology to repress target genes, specific binding sites were necessary for high-efficiency repression. Qi et al. [36] showed that a dCas9 with D10A-H841A mutations arrived 300-fold repression for gene silencing when targeting the non-template DNA strand in *E. coli,* and Gilbert et al. achieved 53-fold repression efficiency with addition of Mxi1 repressor in eukaryotic cells [36]. However, as mentioned above, the repression efficiency in *Y. lipolytica* only arrived 3.1-fold with dCpf1-gRNA complex and 4.5-fold with dCas9-gRNA complex, which was pretty low compared with other strains. Furthermore, it seemed impossible to achieve great repression efficiency only by a simple target position in *Y. lipolytica* based on results above, but extra target sites screening often mandated a significant investment of time and effort. Therefore, developing a convenient multiplex targeting system in *Y. lipolytica* would be useful.

**Fig. 4:**
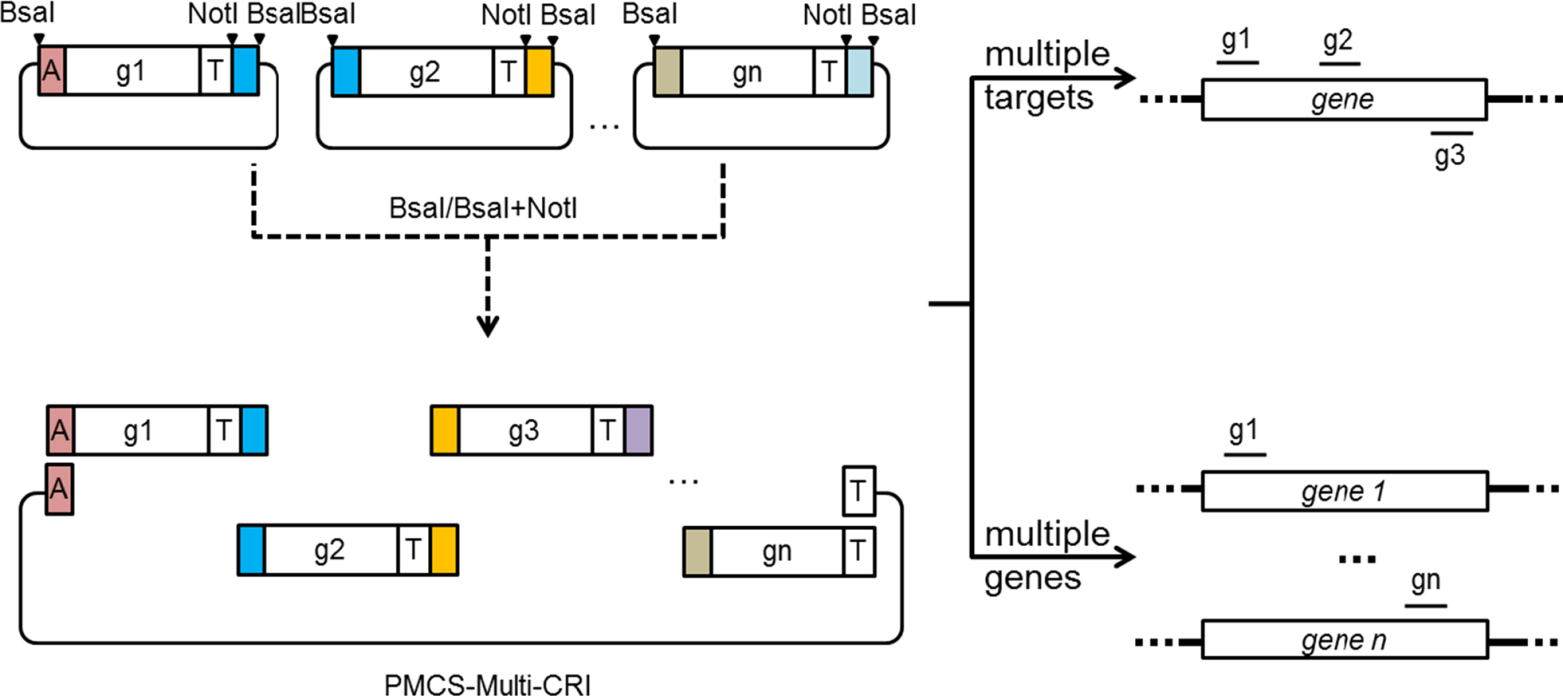
An overview of the Golden-Brick assembly protocol. Multiplex CRISPRi system contains two main parts, one is JLPC/N-n (or JLRC/N-n) plasmid containing gRNA secretion cassette enabling spacers to be ligated into, the other is PMCS-Multi-CRI vector (classified as dCpf1-Multi and dCas9-Multi) containing the gblock of ‘A’ overhang and ‘T’ overhang enabling to assemble various gRNA secretion cassettes. After being released, these gRNA secretion cassettes were assembled with dCpf1-Multi or dCas9-Multi vector in one step. JLPN-n and JLRN-n plasmids were constructed by replacing synthetic hybrid promoter SCR1′-tRNA^Gly^ (JLPC-n and JLRC-n gRNA expression promoter region) with SNR52′-tRNA^Gly^

**Fig. 5:**
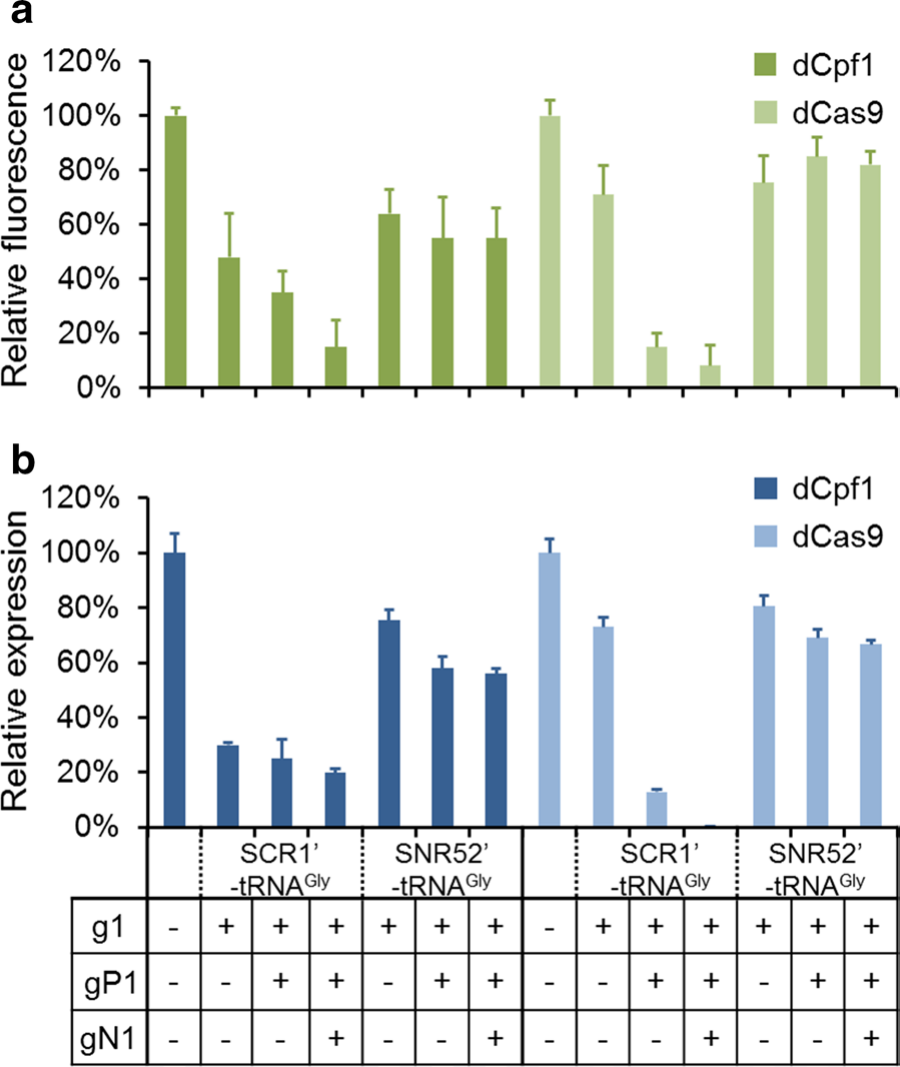
Repression of *gfp* via Multiplex CRISPRi system in *Y. lipolytica*. Regulation of *gfp* expression by dCpf1-Multi and dCas9-Multi system combined with multiplex gRNA targets. **a** Repression of *gfp* by dCpf1-Multi and dCas9-Multi system complexed with single gRNA, double gRNAs and triple gRNAs expressed by SCR1′-tRNA^Gly^ or SNR52′-tRNA^Gly^ promoter. **b** Characterization of the *gfp* gene’s expression level of each strain interfered by dCpf1-Multi and dCas9-Multi system. “+” means possess and “−” means not possess. The error bars (mean ± SD) were derived from triplicate experiments for each strain

In this study, we established a simple and convenient construction tool called multiplex CRISPRi system which provided a manageable strategy to overcome those defects. The robust modular multiplex CRISPRi system could be used for expression of one, two or more customizable transcription units (TU) in a versatile cassette for *Y. lipolytica.* As shown in Fig. 4, the multiplex CRISPRi system contains two main parts, one is JLPC/N-n (or JLRC/N-n) plasmid called transcription units which contains gRNA secretion cassette enabling spacers to be ligated into, and the other is PMCS-Multi-CRI (classified as dCpf1-Multi and dCas9-Multi) vector containing the gBlock of ‘A’ overhang and ‘T’ overhang enabling various gRNA secretion cassettes to be assembled. All TUs were released by BsaI (BsaI and NotI concurrently in the case of the end part) forming two sequential overhangs and assembled with dCpf1-Multi (or dCas9-Multi) vector by Golden-brick assembly method in one step. In this process, once accurate starting fragments were identified, neighboring pairs were ligated together because of the distinctive overhangs. To testify whether the repression efficiency could be improved by multiplex dCas-gRNAs complex in *Y. lipolytica*, we utilized the two multiplex CRISPRi system to target multi locus in *gfp* gene. We randomly selected three targeting sites including the template DNA strand (g1) and the non-template DNA strand (gN1) of the *gfp* coding sequence as well as promoter region (gP1). Then, these three gRNAs were ligated into JLPC-1, JLPC-2 and JLPC-3 plasmid respectively. The forming gRNA secretion cassettes were assembled with dCpf1-Multi vector separately or together to construct single-gRNA, double-gRNA and triple-gRNA repression plasmids. Meanwhile, the dCas9-Multi vector was also assembled with its corresponding gRNA secretion cassettes and formed single-gRNA, double-gRNA and triple-gRNA repression plasmids like the dCpf1-Multi did. We transformed these plasmids into the backbone strain YL-GFP and measured their fluorescence. As shown in Fig. 5a, the single-site (g1) repression efficiency was 52% (~ 2.1-fold) while the double-site (g1-gP1) repression efficiency came to 65% (~ 2.8-fold). And the highest repression efficiency was achieved at 85% (~ 6.7-fold) by using the strategy of three gRNAs towards *gfp* gene simultaneously (g1-gP1-gN1) in the case of dCpf1-Multi. Significantly, the repression efficiency in dCas9-Multi system increased from 29% (g1) to 85% (g1-gP1) and up to 92% (g1-gP1-gN1). Moreover, the mRNA expression levels of influenced *gfp* gene were quantified by RT-qPCR (Fig. 5b), and the transcriptional level was consistent to the fluorescence result in general. These results demonstrated that the multiplex CRISPRi system was very effective to achieve high repression efficiency in *Y. lipolytica* in a short time through rapidly assembling multiplex gRNAs without the need of screening numerous gRNA loci in advance. Additionally, Cory M. Schwartz et al. [31] found another Pol III promoter SNR52′-tRNA^Gly^ could be used to express gRNA in CRISPR/Cas9 system for gene disruption in *Y. lipolytica*, so we designed the JLPN-n and JLRN-n plasmids by replacing synthetic hybrid promoter SCR1′-tRNA^Gly^ (JLPC-n and JLRC-n gRNA expression promoter region) to SNR52′-tRNA^Gly^. To testify whether synthetic promoter SNR52′-tRNA^Gly^ was efficient in repression, several new plasmids were constructed with single gRNA, double gRNAs and triple gRNAs in dCpf1-Multi and dCas9-Multi system respectively, all harboring the SNR52′-tRNA^Gly^ promoter. We transformed them to the strain YL-GFP and measured their respective fluorescence (Fig. 5a). As shown, the single-site (g1) repression efficiency with SNR52′-tRNA^Gly^ promoter was 36 and 25% in dCpf1-Multi and dCas9-Multi system respectively, which was just slightly lower than that of SCR1′-tRNA^Gly^. However, the double-site (g1-gP1) repression efficiency and the triple-site (g1-gP1-gN1) repression efficiency didn’t show obviously increase with the increasing amount of gRNA in both cases, which actually differed from the situation of SCR1′-tRNA^Gly^. The mRNA expression levels of these strains verified the increasing amounts of gRNAs expressed by SNR52′-tRNA^Gly^ promoter didn’t improve the *gfp* repression efficiency (Fig. 5b). Moreover, the *gfp* repression efficiency was 76% with three gRNAs expressed by different promoters, which was also lower than that of only containing SCR1′-tRNA^Gly^ promoter (92%) (Additional file 11: Fig. S5). From the results above, the repression efficiency of SNR52′-tRNA^Gly^ promoter was similar to SCR1′-tRNA^Gly^ promoter in single-site repression while SCR1′-tRNA^Gly^ performed better in the case of multiple-site repression. This study provided a new perspective for single-site repression using SNR52′-tRNA^Gly^ promoter and significantly enhanced expression efficiency via combination of gRNAs expressed by SCR1′-tRNA^Gly^ promoter in *Y. lipolytica.*

Selective and tunable perturbation of gene expression is a fundamental enabling technology in the fields of systems biology and synthetic biology, allowing the design of intricate synthetic circuits and the interrogation of complex natural biological systems [65–67]. Here, we described a multiplex CRISPRi system for multiple gene repression by one-step Golden-brick assembly in the oleochemical-producing yeast *Y. lipolytica*. The Golden-brick assembly method developed in this study provided a simple and more convenient way for plasmid construction than other tools. Biobrick standard assembly requires a step-by-step process using restriction sites and needs four restriction enzyme sites, whereas Golden-brick assembly in this study only needs two restriction enzyme site and all parts can be assembled in one step [68]. Compared with assembly methods based on homologous recombination like Gibson, the Golden-brick assembly method can assemble different parts without the procedure of PCR in advance, which avoids introducing new errors in the process of PCR amplification [69]. In conclusion, multiplex CRISPRi system provided the benefit of improving the regulation efficiency and gave a better strategy to rapidly inhibit target gene expression without the need of screening a large number of target sites in advance in *Y. lipolytica*.

### Multiplex gene interference in *Y. lipolytica*

In metabolic engineering, balancing the expression level of multiple genes is crucial for increasing the productivity of biosynthetic pathways and subsequently for sustainable production of valuable products [57, 67]. As an attractive candidate for industrial biotechnology applications, *Y. lipolytica* has been widely used for production of oleochemicals [8, 14, 70, 71], biofuels [8, 16, 72, 73] and acetyl CoA-derived metabolites [9, 10, 11, 23, 74]. But the library of available tools is not as developed as that of other yeasts such as *Saccharomyces cerevisiae* [34, 35, 54], especially for multiplex gene repression simultaneously [59, 75].

**Fig. 6:**
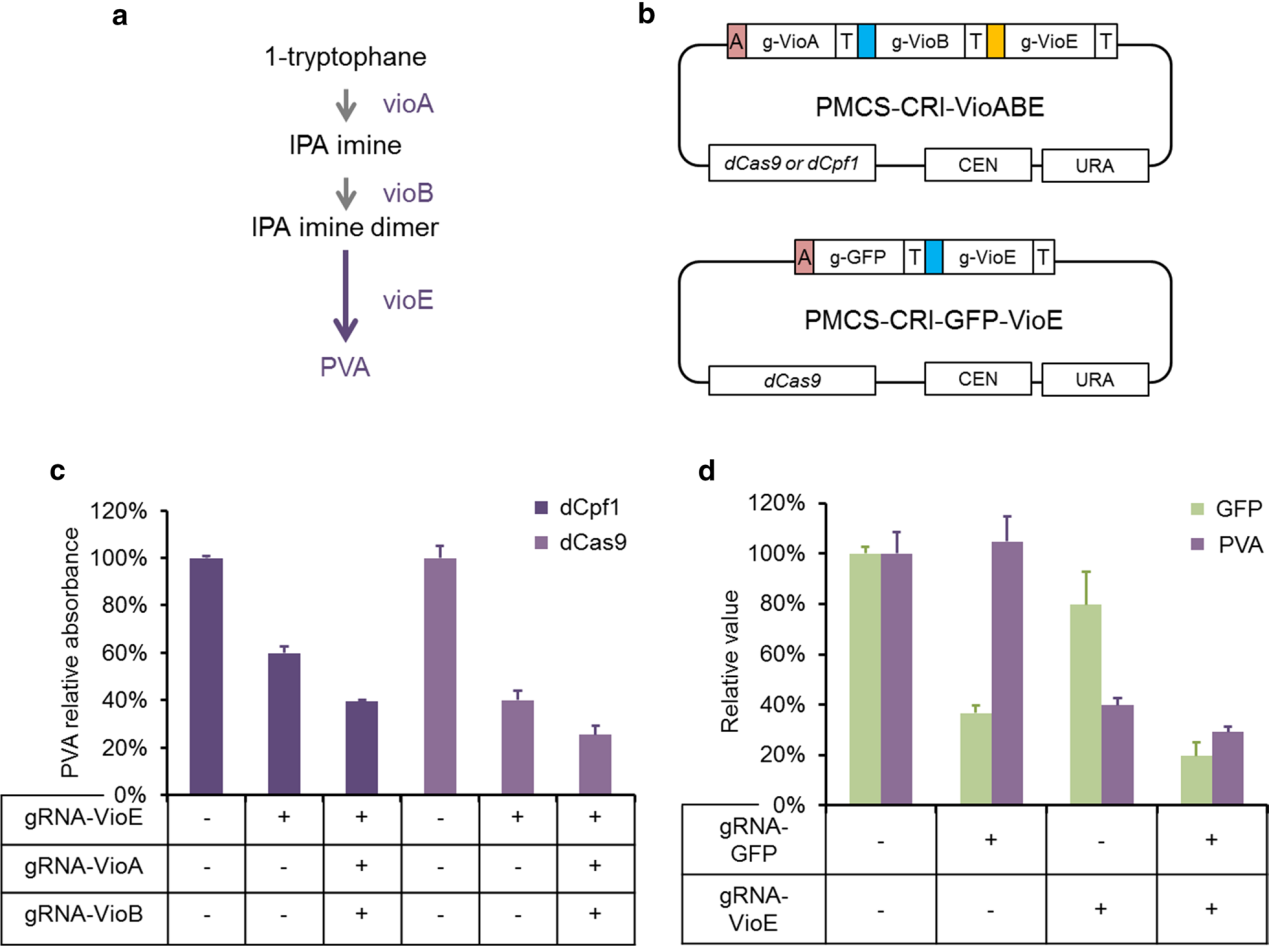
Multiplex gene interference in *Y. lipolytica*. **a** Heterogenous biosynthesis pathway for protodeoxy-violaceinic acid (PVA) production in *Y. lipolytica*. **b** Schematic of the plasmid targeting *vioA*, *vioB* and *vioE* simultaneously. Schematic of plasmid targeting both *gfp* and *vioE*. **c** The PVA relative absorbance of strains interfered by dCpf1-Multi and dCas9-Multi system. **d** The fluorescence and the PVA relative absorbance of strains interfered by dCas9-Multi system. “+” means possess and “−” means not possess. The error bars (mean ± SD) were derived from triplicate experiments for each strain

The multiplex CRISPRi system constructed above had been verified to rapidly repress one gene with high efficiency by combining multiplex gRNAs to different sites of the target gene. In this part, we sought to repress plural genes using the multiplex CRISPRi system in *Y. lipolytica*, testing the efficiency of multiple genes repression. Therefore, we constructed two strains for the purpose of multiplex gene interference. One is VioABE, and the other is VioABE-K8GFP. The construction methods were attached in Additional file 7: Data S2. Strain VioABE contained the protodeoxy-violaceinic acid (PVA) pathway. PVA is a kind of pigment derived from tryptophan and its content can be quantified by relative absorbance of characteristic peak. All of three PVA synthetic pathway gene expression parts which contain *vioA, vioB,* and *vioE* were integrated into rDNA recombined locus to construct strain VioABE (Fig. 6a). We tried to repress *vioA, vioB* and *vioE* genes simultaneously and *vioE* gene solely to test the efficiency of the multiplex CRISPRi system for multiplex gene interference. The reason for choosing *vioE* as the sole target gene was that *vioE* directly biosynthesized prodeoxyviolacein via decarboxylation in PVA synthetic pathway, making the final product with the color of blue-violet. Therefore, dCpf1-Multi vector and dCas9-Multi vector were assembled with its corresponding gRNA secretion cassettes and formed single-gRNA and triple-gRNA repression plasmids (Fig. 6b), all harboring the SCR1′-tRNA^Gly^ promoter. We transformed these four plasmids to the strain VioABE and measured their PVA relative absorbance. As Fig. 6c shown, when repressing *vioE* only, the PVA relative absorbance reduced to 60% and 40% with dCpf1 protein and dCas9 protein separately compared with their corresponding control strains. While the repression of *vioA, vioB,* and *vioE* simultaneously led to further decline in the PVA relative absorbance, 61% and 75% lower than the control respectively, which verified the multiplex CRISPRi system was feasible to implement multiple gene repression in *Y. lipolytica*. VioABE-K8GFP strain was constructed by inserting *gfp* gene controlled by FBA1 intron (FBAint) promoter into the *ku80* locus of the strain VioABE genome (Additional file 7: Data S2). Two gRNAs which targeting *gfp* (gNP1 targeted FBAint promoter) and *vioE* (gP2 targeted GPD promoter) were designed and ligated into JLRC-1 and JLRC-2 plasmids respectively. After being released, the corresponding gRNA secretion cassettes were assembled with dCas9-Multi vector with different kinds of combination and formed four plasmids of Multi-g0, Multi-GFP, Multi-PVA and Multi-GFP-PVA (Fig. 6b). Then the four recombinant plasmids were transformed into strain VioABE-K8GFP respectively. The fluorescence and PVA relative absorbance of these four strains were measured respectively (Fig. 6d) and photos of these interferred strains were shown in Additional file 12: Fig. S6. From the results, we can deduce that every gRNA had repressed its own target gene whether the multiplex CRISPRi system contains one gRNA only or two gRNAs simultaneously. As shown, when repressing *gfp* only, the fluorescence reduced to 37% while there was no obvious change on the PVA relative absorbance. And when only secreting gRNA targeting *vioE*, the PVA relative absorbance reduced to 40% compared with the control. Nevertheless, both PVA relative absorbance and fluorescence decreased when the dCas9-Multi plasmid contained gRNAs targeting both *gfp* and *vioE*. As for why there was more repression of both *gfp* and *vioE* when two gRNA were used together comparing the separately using, we speculated the reason might include the off-target [76] of gRNA and global repression induced by the CRISPRi as well as local metabolism environment of *Y. lipolytica* [77].

In most cases, for multiplex gene regulation or epigenetic modifications, multiple gRNAs may need to be independently expressed, and the construction procedure is time-consuming [69]. By using a multiplexed gRNA targeting strategy, we achieved efficient transcriptional simultaneous repression of several targeted genes in one step. In this study, we have demonstrated that the multiplex CRISPRi system could be used for PVA and GFP regulation solely or simultaneously, which promised to be a potent transformative tool that will be extremely valuable for metabolic engineering requiring throttled flux through essential pathways in *Y. lipolytica*.

## Conclusion

In this work, we demonstrated successful CRISPRi-mediated regulation of gene expression via four different repressors dCpf1, dCas9, dCpf1-KRAB and dCas9-KRAB in *Y. lipolytica.* By using a multiplexed gRNA targeting strategy, efficient transcriptional simultaneous repression of several targeted genes and different sites of one gene was achieved in one step without the need of screening a large number of target sites. This study thus paves a new avenue to facilitate metabolic engineering, synthetic biology and functional genomic studies of *Y. lipolytica*.

## Additional files

**Additional file 1: Table S1.** Strains and plasmids used in this study.

**Additional file 2: Table S2.** Primer sequences.

**Additional file 3: Data S1.** Detailed Plasmid Assembly Protocol.

**Additional file 4: Fig. S1.** The relationship between PVA content and Relative absorbance.

**Additional file 5: Table S3.** Primer sequences for RT-PCR.

**Additional file 6: Fig. S2.** GFP-based reporter system in *Y. lipolytica.* (a) Selection of functional reporter system in *Y. lipolytica*. The fluorescence levels of RedStar2, YFP and sfGFP driven by the TEFin promoter in *Y. lipolytica* were tested by multi-mode microplate reader. (b) A synthetic fluorescence-based reporter system containing an *sfGFP* gene is inserted into the *Y. lipolytica* genome (the rDNA locus). The mean fluorescence data were collected by multi-mode microplate reader analysis for comparison at a time point of 48 h. The control(con) for these experiments was *Y. lipolytica* strain without any gene integrated into. The error bars (mean ± SD) were derived from triplicate experiments for each strain.

**Additional file 7: Data S2.** The details for constructing strain VioABE, YL-GFP and VioABE-K8GFP.

**Additional file 8: Fig. S3.** Repression of *gfp* in *Y. lipolytica* by dCas9-Mxi1. The Mxi1 repressor was fused to the C-terminus of dCas9 and formed the plasmid PMCS-dCas9-Mxi1. CRISPRi repression of *gfp* with dCas9-Mxi1 complexed with ten gRNAs targeting different regions. The control (g0) shows fluorescence of the cells with dCas9-Mxi1 protein but without the gRNA. The error bars (mean ± SD) were derived from triplicate experiments for each strain.

**Additional file 9: Fig. S4.** Repression and disruption of *pex10* in *Y. lipolytica* by dCas9 and Cas9. (a) Characterization of the *pex10* gene’s repression level of six strains interfered by dCas9. (b) Disruption rates of *pex10* after 4 days of outgrowth in selective liquid media with different transformed CRISPR-Cas9 plasmids. (c) Placement of gRNA protospacers on the target *pex10* gene. (d) Phenotype of *pex10* disruptants. Six plates screening of *pex10* disrupted phenotypes on SC-Ura and SCO-Ura media. The error bars (mean ± SD) were derived from triplicate experiments for each strain.

**Additional file 10: Data S3.** The details for constructing Cas9-pex10 plasmids and transformation method.

**Additional file 11: Fig. S5.** Repression of *gfp* in *Y. lipolytica* by dCas9-Multi. Efficiency of various gRNA secretion cassettes with different synthetic hybrid promoters on gene repression. The error bars (mean ± SD) were derived from triplicate experiments for each strain.

**Additional file 12: Fig. S6.** Photos and its corresponding microscopic images of the interfered strains by dCas9-Multi system. Four related plasmids were transformed into strain VioABE-K8GFP respectively to form four strains: control (means no gRNA towards *gfp* or *vioE*, only backbone of plasmid), gRNA-GFP (The dCas9-Multi plasmid only contains gRNA targeting *gfp*), gRNA-PVA (The dCas9-Multi plasmid only contains gRNA targeting *vioE*), gRNA-GFP-PVA (The dCas9-Multi plasmid contains gRNAs both targeting *gfp* and *vioE*).
