# Supplementary material for: Gene repression via multiplex gRNA strategy in Y. lipolytica
Source: Microb Cell Fact. 2018 Apr 20;17:62. doi: 10.1186/s12934-018-0909-8 (PMC5910576; doi:10.1186/s12934-018-0909-8)
Supplement: Supplementary file 1 — Additional file 1: Table S1. Strains and plasmids used in this study. [file 12934_2018_909_MOESM1_ESM.docx]

**Table S1. Strains and plasmids used in this study**

| Strains or plasmids | Characteristics | Source or reference |
| --- | --- | --- |
| Strains |  |  |
| Yarrowia lipolytica  ATCC 201249 | *MATA ura3-302 leu2-270 lys8-11 PEX17-HA , Ref* | Wickerham et al |
| Yl-GFP | ATCC201249 r*DNA:: EXP1p-sfGFP-xpr2t-Hph* | This work |
| VioABE | ATCC201249  *rDNA::EXP1p-VioA-xpr2t-TEF1p-VioB-lip2t-GPDp-VioE-OCTt-Hph* | This work |
| VioABE-K8GFP | VioABE  *△ku80::FBAinp-sfGFP-OCTt-Ura* | This work |
| Plasmids |  |  |
| PMCS -ura | *URA3* guide RNA module expression cassette in pMCSCen1 | GenScript |
| PMCS-gRNAc-ura | *URA3* guide RNA module expression cassette in pMCSCen1 | GenScript |
| PMCS-dCas9 | *URA3* guide RNA module and dCas9 expression cassette in pMCSCen1 | GenScript |
| PMCS-dCpf1 | *URA3* guide RNA module and dCpf1 expression cassette in pMCSCen1 | GenScript |
| dCas9-Multi | *URA3* guide RNA module GoldenBrick assembly site linker A(AGGT)-doule BsaI-linker n(GGCC same as NotI) and dCas9 expression cassette in pMCSCen1 | GenScript |
| dCpf1-Multi | *URA3* guide RNA module GoldenBrick assembly site linker A(AGGT)-doule BsaI-linker n(GGCC same as NotI) and dCpf1 expression cassette in pMCSCen1 | GenScript |
| PMCS-dCas9-KRAB | *URA3* guide RNA module and dCas9-KRAB expression cassette in pMCSCen1 | GenScript |
| PMCS-dCas9-MXI1 | *URA3* guide RNA module and dcas9-MXI1 expression cassette in pMCSCen1 | GenScript |
| PMCS-dCpf1-KRAB | *URA3* guide RNA module and dCpf1-KRAB expression cassette in pMCSCen1 | GenScript |
| JLRC -1 | guide RNA module controlled by synthetic hybrid promoter SCR-tRNA^Gly^ with GoldenBrick linker A(AGGT) on its head and NotI-linker B(CCAC) on its end | GenScript |
| JLRC -2 | guide RNA module controlled by synthetic hybrid promoter SCR-tRNA^Gly^ with GoldenBrick linker B(CCAC) on its head and NotI-linker C(GCTT) on its end | GenScript |
| JLRC -3 | guide RNA module controlled by synthetic hybrid promoter SCR-tRNA^Gly^ with GoldenBrick linker C(GCTT) on its head and NotI-linker D(TACG) on its end | GenScript |
| JLRN-1 | guide RNA module controlled by synthetic hybrid promoter SNR52-tRNA^Gly^ with GoldenBrick linker A(AGGT) on its head and NotI-linker B(CCAC) on its end | GenScript |
| JLRN-2 | guide RNA module controlled by synthetic hybrid promoter SNR52-tRNA^Gly^ with GoldenBrick linker B(CCAC) on its head and NotI-linker C(GCTT) on its end | GenScript |
| JLRN-3 | guide RNA module controlled by synthetic hybrid promoter SNR52-tRNA^Gly^ with GoldenBrick linker C(GCTT) on its head and NotI-linker D(TACG) on its end | GenScript |
| JLP1C -1 | guide RNA module controlled by synthetic hybrid promoter SCR-tRNA^Gly^ with GoldenBrick linker A(AGGT) on its head and NotI-linker B(CCAC) on its end | GenScript |
| JLPC -2 | guide RNA module controlled by synthetic hybrid promoter SCR-tRNA^Gly^ with GoldenBrick linker B(CCAC) on its head and NotI-linker C(GCTT) on its end | GenScript |
| JLPC -3 | guide RNA module controlled by synthetic hybrid promoter SCR-tRNA^Gly^ with GoldenBrick linker C(GCTT) on its head and NotI-linker D(TACG) on its end | GenScript |
| JLPN-1 | guide RNA module controlled by synthetic hybrid promoter SNR52-tRNA^Gly^ with GoldenBrick linker A(AGGT) on its head and NotI-linker B(CCAC) on its end | GenScript |
| JLPN-2 | guide RNA module controlled by synthetic hybrid promoter SNR52-tRNA^Gly^ with GoldenBrick linker B(CCAC) on its head and NotI-linker C(GCTT) on its end | GenScript |
| JLPN-3 | guide RNA module controlled by synthetic hybrid promoter SNR52-tRNA^Gly^ with GoldenBrick linker C(GCTT) on its head and NotI-linker D(TACG) on its end | GenScript |
|  |  |  |
| PMCS-dCas9-GFP-g1 | PMCS-dCas9 with gfp-g1 gRNA | This work |
| PMCS-dCas9-GFP-g2 | PMCS-dCas9 with gfp-g2 gRNA | This work |
| PMCS-dCas9-GFP-g3 | PMCS-dCas9 with gfp-g3 gRNA | This work |
| PMCS-dCas9-GFP-gN1 | PMCS-dCas9 with gfp-gN1 gRNA | This work |
| PMCS-dCas9-GFP-gN2 | PMCS-dCas9 with gfp-gN2 gRNA | This work |
| PMCS-dCas9-GFP-gN3 | PMCS-dCas9 with gfp-gN3 gRNA | This work |
| PMCS-dCas9-GFP-gP1 | PMCS-dCas9 with gfp-gP1 gRNA | This work |
| PMCS-dCas9-GFP-gP2 | PMCS-dCas9 with gfp-gP2 gRNA | This work |
| PMCS-dCas9-GFP-gNP1 | PMCS-dCas9 with gfp-gNP1 gRNA | This work |
| PMCS-dCas9-GFP-gNP2 | PMCS-dCas9 with gfp-gNP2 gRNA | This work |
| PMCS-dCpf1-GFP-g1 | PMCS-dCpf1 with gfp-g1 gRNA | This work |
| PMCS-dCpf1-GFP-g2 | PMCS-dCpf1 with gfp-g2 gRNA | This work |
| PMCS-dCpf1-GFP-g3 | PMCS-dCpf1 with gfp-g3 gRNA | This work |
| PMCS-dCpf1-GFP-gN1 | PMCS-dCpf1 with gfp-gN1 gRNA | This work |
| PMCS-dCpf1-GFP-gN2 | PMCS-dCpf1 with gfp-gN2 gRNA | This work |
| PMCS-dCpf1-GFP-gN3 | PMCS-dCpf1 with gfp-gN3 gRNA | This work |
| PMCS-dCpf1-GFP-gP1 | PMCS-dCpf1 with gfp-gP1 gRNA | This work |
| PMCS-dCpf1-GFP-gP2 | PMCS-dCpf1 with gfp-gP2 gRNA | This work |
| PMCS-dCpf1-GFP-gNP1 | PMCS-dCpf1 with gfp-gNP1 gRNA | This work |
| PMCS-dCpf1-GFP-gNP2 | PMCS-dCpf1 with gfp-gNP2 gRNA | This work |
| PMCS-dCas9-KRAB-GFP-g1 | PMCS-dCas9-KRAB with gfp-g1 gRNA | This work |
| PMCS-dCas9-KRAB-GFP-g2 | PMCS-dCas9-KRAB with gfp-g2 gRNA | This work |
| PMCS-dCas9-KRAB-GFP-g3 | PMCS-dCas9-KRAB with gfp-g3 gRNA | This work |
| PMCS-dCas9-KRAB-GFP-gN1 | PMCS-dCas9-KRAB with gfp-gN1 gRNA | This work |
| PMCS-dCas9-KRAB-GFP-gN2 | PMCS-dCas9-KRAB with gfp-gN2 gRNA | This work |
| PMCS-dCas9-KRAB-GFP-gN3 | PMCS-dCas9-KRAB with gfp-gN3 gRNA | This work |
| PMCS-dCas9-KRAB-GFP-gP1 | PMCS-dCas9-KRAB with gfp-gP1 gRNA | This work |
| PMCS-dCas9-KRAB-GFP-gP2 | PMCS-dCas9-KRAB with gfp-gP2 gRNA | This work |
| PMCS-dCas9-KRAB-GFP-gNP1 | PMCS-dCas9-KRAB with gfp-gNP1 gRNA | This work |
| PMCS-dCas9-KRAB-GFP-gNP2 | PMCS-dCas9-KRAB with gfp-gNP2 gRNA | This work |
| PMCS-dCas9-MXI1-GFP-g1 | PMCS-dCas9-KRAB with gfp-g1 gRNA | This work |
| PMCS-dCas9-MXI1-GFP-g2 | PMCS-dCas9-MXI1 with gfp-g2 gRNA | This work |
| PMCS-dCas9-MXI1-GFP-g3 | PMCS-dCas9-MXI1 with gfp-g3 gRNA | This work |
| PMCS-dCas9-MXI1-GFP-gN1 | PMCS-dCas9-MXI1 with gfp-gN1 gRNA | This work |
| PMCS-dCas9-MXI1-GFP-gN2 | PMCS-dCas9-MXI1 with gfp-gN2 gRNA | This work |
| PMCS-dCas9-MXI1-GFP-gN3 | PMCS-dCas9-MXI1 with gfp-gN3 gRNA | This work |
| PMCS-dCas9-MXI1-GFP-gP1 | PMCS-dCas9-MXI1 with gfp-gP1 gRNA | This work |
| PMCS-dCas9-MXI1-GFP-gP2 | PMCS-dCas9-MXI1 with gfp-gP2 gRNA | This work |
| PMCS-dCas9-MXI1-GFP-gNP1 | PMCS-dCas9-MXI1 with gfp-gNP1 gRNA | This work |
| PMCS-dCas9-MXI1-GFP-gNP2 | PMCS-dCas9-MXI1 with gfp-gNP2 gRNA | This work |
| PMCS-dCpf1-KRAB-GFP-g1 | PMCS-dCpf1-KRAB with gfp-g1 gRNA | This work |
| PMCS-dCpf1-KRAB-GFP-g2 | PMCS-dCpf1-KRAB with gfp-g2 gRNA | This work |
| PMCS-dCpf1-KRAB-GFP-g3 | PMCS-dCpf1-KRAB with gfp-g3 gRNA | This work |
| PMCS-dCpf1-KRAB-GFP-gN1 | PMCS-dCpf1-KRAB with gfp-gN1 gRNA | This work |
| PMCS-dCpf1-KRAB-GFP-gN2 | PMCS-dCpf1-KRAB with gfp-gN2 gRNA | This work |
| PMCS-dCpf1-KRAB-GFP-gN3 | PMCS-dCpf1-KRAB with gfp-gN3 gRNA | This work |
| PMCS-dCpf1-KRAB-GFP-gP1 | PMCS-dCpf1-KRAB with gfp-gP1 gRNA | This work |
| PMCS-dCpf1-KRAB-GFP-gP2 | PMCS-dCpf1-KRAB with gfp-gP2 gRNA | This work |
| PMCS-dCpf1-KRAB-GFP-gNP1 | PMCS-dCpf1-KRAB with gfp-gNP1 gRNA | This work |
| PMCS-dCpf1-KRAB-GFP-gNP2 | PMCS-dCpf1-KRAB with gfp-gNP2 gRNA | This work |
| C01-GFP-g1 | JLRC-1 with gfp-g1 gRNA | This work |
| C02-GFP-gP1 | JLRC-2 with gfp-gP1 gRNA | This work |
| C03-GFP-gN1 | JLRC-3 with gfp-gN1 gRNA | This work |
| N01-GFP-g1 | JLRN-1 with gfp-g1 gRNA | This work |
| N02-GFP-gP1 | JLRN-2 with gfp-gP1 gRNA | This work |
| N03-GFP-gN1 | JLRN-3 with gfp-gN1 gRNA | This work |
| JLPC01-GFP-g1 | JLPC-1 with gfp-g1 gRNA | This work |
| JLPC0 2-GFP-gP1 | JLPC-2 with gfp-gP1 gRNA | This work |
| JLPC03-GFP-gN1 | JLPC-3 with gfp-gN1 gRNA | This work |
| JLPN01-GFP-g1 | JLPN-1 with gfp-g1 gRNA | This work |
| JLPN02-GFP-gP1 | JLPN-2 with gfp-gP1 gRNA | This work |
| JLPN03-GFP-gN1 | JLPN-3 with gfp-gN1 gRNA | This work |
| Multi-gfp-dCas9-g0 | dCas9 expression cassette in PMCS-Multi | This work |
| Multi-gfp-C-g1 | dCas9-Multi with JLRC-1-gfp-g1 gRNA | This work |
| Multi-gfp-C-g1-gp1 | dCas9-Multi with JLRC-1-gfp-g1 and JLRC-2-gfp-gp1 gRNA | This work |
| Multi-gfp-C-g1-gp1-gN1 | dCas9-Multi with JLRC-1-gfp-g1, JLRC-2-gfp-gp1 and JLRC-3-gfp-gN1, gRNA | This work |
| Multi-gfp-N-g1 | dCas9-Multi with JLRN-1-gfp-g1 gRNA | This work |
| Multi-gfp-N-g1-gp1 | dCas9-Multi with JLRN-1-gfp-g1 and JLRN-2-gfp-gp1 gRNA | This work |
| Multi-gfp-N-g1-gp1-gN1 | dCas9-Multi with JLRN-1-gfp-g1, JLRN-2-gfp-gp1 and JLRN-3-gfp-gN1, gRNA | This work |
| Multi-gfp-N-g1-C-gp1-gN1 | dCas9-Multi with JLRN-1-gfp-g1, JLRC-2-gfp-gp1 and JLRC-3-gfp-gN1, gRNA |  |
| Multi-gfp-dCpf1-g0 | dCpf1 expression cassette in PMCS-Multi | This work |
| Cpf1-Multi-gfp-C-g1 | dCPF1-Multi with JLPC-1-gfp-g1 gRNA | This work |
| Cpf1-Multi-gfp-C-g1-gp1 | dCPF1-Multi with JLPC-1-gfp-g1 and JLPC-2-gfp-gP1 gRNA | This work |
| Cpf1-Multi-gfp-C-g1-gN1 | dCPF1-Multi with JLPC-1 gfp-g1 and JLPC-2-gfp-gP1 and JLPC-3-gfp-gN1 gRNA | This work |
| Cpf1-Multi-gfp-N-g1 | dCPF1-Multi with JLPN-1-gfp-g1 gRNA | This work |
| Cpf1-Multi-gfp-N-g1-gp1 | dCPF1-Multi with JLPN-1-gfp-g1 and JLPN-2-gfp-gP1 gRNA | This work |
| Cpf1-Multi-gfp-N-g1-gN1 | dCPF1-Multi with JLPN-1 gfp-g1 and JLPN-2-gfp-gP1 and JLPN-3-gfp-gN1 gRNA | This work |
| C01-VioE | JLRC-1 with vioE-gP2 gRNA | This work |
| C01-VioA | JLRC-1 with vioA-g1 gRNA | This work |
| C02-VioB | JLRC-2 with vioB-g1 gRNA | This work |
| C03-VioE | JLRC-3 with vioE-gP2 gRNA | This work |
| JLPC01-VioE | JLPC-1 with vioE-gP2 gRNA | This work |
| JLPC01-VioA | JLPC-1 with vioA-g1 gRNA | This work |
| JLPC02-VioB | JLPC-2with vioB-g1 gRNA | This work |
| JLPC03-VioE | JLPC-3 with vioE-gP2 gRNA | This work |
| Multi-vioE-C-gP2 | dCas9-Multi with JLRC-1-vioE-gP2 gRNA | This work |
| Multi-vioABE-C-g1-g1-gp2 | dCas9-Multi with JLRC-1-vioA-g1 JLRC-2-vioB-g1 and JLRC-3-vioE-gP2 gRNA | This work |
| Cpf1-Multi-vioE-C-gP2 | dCpf1-Multi with JLPC-1-vioE-gP2 gRNA | This work |
| Cpf1-Multi-vioABE-C-g1-g1-gp2 | dCpf1-Multi with JLPC-1-vioA-g1, JLPC-2-vioB-g1 and JLPC-3-vioE-gP2 gRNA | This work |
|  |  |  |
| C01-GFP-gNP1 | JLRC-1 with gfp-gNP1 gRNA | This work |
|  |  |  |
| C02-VioE-gP2 | JLRC-2 with vioE-gP2 gRNA | This work |
| Multi -GFP | PMCS-Multi with JLRC-1- gfp-gNP1 gRNA | This work |
| Multi -PVA | PMCS-Multi with JLRC-2-VioE-gP2 gRNA | This work |
| Multi- GFP-PVA | PMCS-Multi with JLRC-1-gfp-gNP1 and JLRC-2-VioE-gP2 gRNA | This work |
| Cas9-pex10-g1 | Cas9 expression cassette in pMCSCen1 with pex10-g1 gRNA | This work |
| Cas9-pex10-g2 | Cas9 expression cassette in pMCSCen1 with pex10-g2 gRNA | This work |
| Cas9-pex10-g3 | Cas9 expression cassette in pMCSCen1 with pex10-g3 gRNA | This work |
| Cas9-pex10-gN1 | Cas9 expression cassette in pMCSCen1 with pex10-gN1 gRNA | This work |
| Cas9-pex10-gN 2 | Cas9 expression cassette in pMCSCen1 with pex10-gN2 gRNA | This work |
| Cas9-pex10-gN3 | Cas9 expression cassette in pMCSCen1 with pex10-gN3 gRNA | This work |
| PMCS-dCas9-pex10-g1 | PMCS-dCas9 with pex10-g1 gRNA | This work |
| PMCS-dCas9-pex10-g2 | PMCS-dCas9 with pex10-g2 gRNA | This work |
| PMCS-dCas9-pex10-g3 | PMCS-dCas9 with pex10-g3 gRNA | This work |
| PMCS-dCas9-pex10-gN1 | PMCS-dCas9 with pex10-gN1 gRNA | This work |
| PMCS-dCas9-pex10-gN2 | PMCS-dCas9 with pex10-gN2 gRNA | This work |
| PMCS-dCas9-pex10-gN3 | PMCS-dCas9 with pex10-gN3 gRNA | This work |
