# Supplementary material for: Gene repression via multiplex gRNA strategy in Y. lipolytica
Source: Microb Cell Fact. 2018 Apr 20;17:62. doi: 10.1186/s12934-018-0909-8 (PMC5910576; doi:10.1186/s12934-018-0909-8)
Supplement: Supplementary file 2 — Additional file 2: Table S2. Primer sequences. [file 12934_2018_909_MOESM2_ESM.docx]

**Table S2. Primer sequences**

| Name | Sequence |
| --- | --- |
| sfGFP-cas9-gNP2-F | ACGT ATTTGTACGCGGTGGACAAC |
| sfGFP-cas9-gNP2-R | AAAC GTTGTCCACCGCGTACAAAT |
| sfGFP-cas9-gP2-F | ACGT GTCCACCGCGTACAAATGTT |
| sfGFP-cas9-gP2-R | AAAC AACATTTGTACGCGGTGGAC |
| sfGFP-cas9-gNP1-F | ACGT CTTGTTTGTGTTTGTGCAAG |
| sfGFP-cas9-gNP1-R | AAAC CTTGCACAAACACAAACAAG |
| sfGFP-cas9-gP1-F | ACGT ACAAACAAGCATTATATATA |
| sfGFP-cas9-gP1-R | AAAC TATATATAATGCTTGTTTGT |
| sfGFP-cas9-g1-F | ACGT ACAGCAAATGCGAAAGGGTG |
| sfGFP-cas9-g1-R | AAAC CACCCTTTCGCATTTGCTGT |
| sfGFP-cas9-Ng1-F | ACGT CACCAGGATGGGCACCACAC |
| sfGFP-cas9-Ng1-R | AAAC GTGTGGTGCCCATCCTGGTG |
| sfGFP-cas9-g2-F | ACGT GGAGCGAACCATCTCCTTCA |
| sfGFP-cas9-g2-R | AAAC TGAAGGAGATGGTTCGCTCC |
| sfGFP-cas9-Ng2-F | ACGT CACCTCGGCTCGGGTCTTGT |
| sfGFP-cas9-Ng2-R | AAAC ACAAGACCCGAGCCGAGGTG |
| sfGFP-cas9-g3-F | ACGT CAACGAGAAGCGAGATCATA |
| sfGFP-cas9-g3-R | AAAC TATGATCTCGCTTCTCGTTG |
| sfGFP-cas9-Ng3-F | ACGT TATGATCTCGCTTCTCGTTG |
| sfGFP-cas9-Ng3-R | AAAC CAACGAGAAGCGAGATCATA |
| sfGFP-cpf1-gP2-F | AGAT ATCGAGCCCTAACCCCTGCC |
| sfGFP-cpf1-gP2-R | AAAA GGCAGGGGTTAGGGCTCGAT |
| sfGFP-cpf1-NP2-F | AGAT CCTGATGGGCAGGGGTTAGG |
| sfGFP-cpf1-NP2-R | AAAA CCTAACCCCTGCCCATCAGG |
| sfGFP-cpf1-gP1-F | AGAT CACACAAGACATATCTACAG |
| sfGFP-cpf1-gP1-R | AAAA CTGTAGATATGTCTTGTGTG |
| sfGFP-cpf1-NP1-F | AGAT CTGTAGATATGTCTTGTGTG |
| sfGFP-cpf1-NP1-R | AAAA CACACAAGACATATCTACAG |
| sfGFP-cpf1-g1-F | AGAT ACCGGTGTGGTGCCCATCCT |
| sfGFP-cpf1-g1-R | AAAA AGGATGGGCACCACACCGGT |
| sfGFP-cpf1-Ng1-F | AGAT ACGTCGCCGTCCAGCTCCAC |
| sfGFP-cpf1-Ng1-R | AAAA GTGGAGCTGGACGGCGACGT |
| sfGFP-cpf1-Ng2-F | AGAT TAGGTGCCGTCGTCCTTGAA |
| sfGFP-cpf1-Ng2-R | AAAA TTCAAGGACGACGGCACCTA |
| sfGFP-cpf1-g2-F | AGAT AAGGACGACGGCACCTACAA |
| sfGFP-cpf1-g2-R | AAAA TTGTAGGTGCCGTCGTCCTT |
| sfGFP-cpf1-g3-F | AGAT AAGATCCGACACAACGTCGA |
| sfGFP-cpf1-g3-R | AAAA TCGACGTTGTGTCGGATCTT |
| sfGFP-cpf1-Ng3-F | AGAT TGCTGGTAGTGGTCGGCCAG |
| sfGFP-cpf1-Ng3-R | AAAA CTGGCCGACCACTACCAGCA |
| Pex10-cas9-g1-F | ACGTCTGCCTCGGCTCAAAATGTG |
| Pex10-cas9-g1-R | AAACCACATTTTGAGCCGAGGCAG |
| Pex10-cas9-g2-F | ACGTTGACACTACAACTACACACC |
| Pex10-cas9-g2-R | AAACGGTGTGTAGTTGTAGTGTCA |
| Pex10-cas9-g3-F | ACGTCTTGTGGGCTCCAAGACTCT |
| Pex10-cas9-g3-R | AAAC AGAGTCTTGGAGCCCACAAG |
| Pex10-cas9-Ng1-F | ACGT ATTGTCGCTCATGTTGGACC |
| Pex10-cas9-Ng1-R | AAACGGTCCAACATGAGCGACAAT |
| Pex10-cas9-Ng2-F | ACGTGTACTCTTCTCCGAGAGTCT |
| Pex10-cas9-Ng2-R | AAACAGACTCTCGGAGAAGAGTAC |
| Pex10-cas9-Ng3-F | ACGTCCCGCTCCTTCCATGTTTCC |
| Pex10-cas9-Ng3-R | AAACGGAAACATGGAAGGAGCGGG |
| VioE-cas9-gp2-F | ACGT TCCAAGGCAACATTTATATA |
| VioE-cas9-gp2-R | AAAC TATATAAATGTTGCCTTGGA |
| VioE-cpf1-gp2-F | AGATTATAAGGGTCTGCATCGCCG |
| VioE-cpf1-gp2-R | AAAACGGCGATGCAGACCCTTATA |
| VioA-cas9-g1-F | ACGTGATATCTGCATTGTTGGTGC |
| VioA-cas9-g1-R | AAACGCACCAACAATGCAGATATC |
| VioB-cas9-g1-F | ACGTCGCGTATCCACTTCCGTGGC |
| VioB-cas9-g1-R | AAACGCCACGGAAGTGGATACGCG |
| VioA-cpf1-g1-F | AGATTTCCGATATCTGCATTGTTG |
| VioA-cpf1-g1-R | AAAACAACAATGCAGATATCGGAA |
| VioB-cpf1-g1-F | AGAT TGGATTTCCCGCGTATCCAC |
| VioB-cpf1-g1-R | AAAAGTGGATACGCGGGAAATCCA |
| PMCS-test-F： | ACCACCACTGCACTACCACTACACC |
| sfGFP-cas9- F8-in-gNP1-F | ACGTAAAGAGACTGGCAACCGATT |
| sfGFP-cas9- F8-in-gNP1-R | AAACAATCGGTTGCCAGTCTCTTT |
| dcpf1-R | TCAGGTTCTGGTTGAACAGGTTC |
| TEFin-273-F | GTGGTTGGGACTTTAGCCAAGG |
| dcas9-R | GAACTTAATCATGTGGGCCAGAG |
| N通引-70-F | AGGTAATTTACCCTAGCCCATTGTC |
| JLRC通引-59-F | GAATCATAACAAGGGTTAGGGTGTG |
| Cas9-F | ATGGATAAGAAATACTCCATTGGCCTG |
| Cas9-R | GATGGCGTCAGACAGGTTCTTGG |
| BsaI-K8-GFP-F | GGTCTCCCCAGCGAAAGGGTGAGGAGCTGTTC |
| BsaI-K8-GFP-R | GGTCTCCTTTACTTGTACAGCTCGTCCATACCGTG |
