# Supplementary material for: Gene repression via multiplex gRNA strategy in Y. lipolytica
Source: Microb Cell Fact. 2018 Apr 20;17:62. doi: 10.1186/s12934-018-0909-8 (PMC5910576; doi:10.1186/s12934-018-0909-8)
Supplement: Supplementary file 3 — Additional file 3: Data S1. Detailed Plasmid Assembly Protocol. [file 12934_2018_909_MOESM3_ESM.docx]

**Additional file 3: Data. S1**

**Detailed Plasmid Assembly Protocol**1. All guide RNA cassettes plasmids (contain PMCS-dCas9, PMCS-dCpf1, PMCS-dCas9-KRAB, PMCS-dCas9-MXI1, JLRC-1, JLRN-1, JLRC-2, JLRC-3) were assembled using the following protocol according to Zhao, H (2014):
1) Select a 20 nt protospacer of interest.

1. Design two 24 nt oligonucleotides (4 nt 5’ sticky end + 20 nt spacer sequence) with the sticky ends **AGAT** on the forward primer and AAAA on the reverse primer, when inhibitor was dcpf1 and **ACGT** on the forward primer and AAAC on the reverse primer, when inhibitor was dcas9. For example, if the spacer sequence is ATTTGTACGCGGTGGACAAC and the inhibitor is dcas9, then the two primers are:
   a. Spacer-for: 5’-ACGT ATTTGTACGCGGTGGACAAC-3’
   b. Spacer-rev: 5’-AAAC GTTGTCCACCGCGTACAAAT-3’
2. For single spacers, anneal spacer oligos as follows:
   a. Resuspend both oligos to 100µM in water
   b. Mix 2uL FOR + 2uL REV + 36uL 30mM HEPES, pH 7.8
   c. Heat to 95°C for 5min, then ramp to 4°C at 0.1°C/sec

d.Add 120uL water to the reaction system
4) Insert annealed spacer by Golden Gate assembly.
Golden Gate reaction mixture:
Backbone X µL 100 ng
Insert 0.5 µL From diluted annealed oligo stock

T4 Ligase Buffer (NEB) 2 µL
T4 ligase (NEB) 1 µL 400 U/µL stock is sufficient; add last
BbsI (NEB) 1 µL Stored at -80 °C
H2O Y µL
 20 µL

2. For plasmid PMCS-Multi, one or more guide RNA cassettes were linked to it. The Golden Gate reaction mixture as followed:

Backbone X µL 100ng
cassette1 X1 µL

cassette2 X2 µL equal number of moles

Cassette3 X3 µL

T4 Ligase Buffer (NEB) 2 µL
T4 ligase (NEB) 1 µL 400 U/µL stock is sufficient; add last
BbsI (NEB) 1 µL Stored at -80 °C
H2O Y µL
 20 µL

Golden Gate Program: 37°C 10 min
 16°C 10 min
 Goto step 1, 9 times
 50°C, 5 min
 65°C, 20 min
 4°C, forever
5) Transform 5 to 20 µL of each reaction to E. coli Trans T1 by heat shock (manufacturer’s protocol)
6) Plate 10% of recovery culture on selective plates with 8uL of 0.5 M IPTG and 40uL of
25mg/mL X-gal (in DMSO).
7) Pick white colonies to selective LB and recover plasmid.
