# Supplementary figures and images for: Gene repression via multiplex gRNA strategy in Y. lipolytica
Source: Microb Cell Fact. 2018 Apr 20;17:62. doi: 10.1186/s12934-018-0909-8 (PMC5910576; doi:10.1186/s12934-018-0909-8)

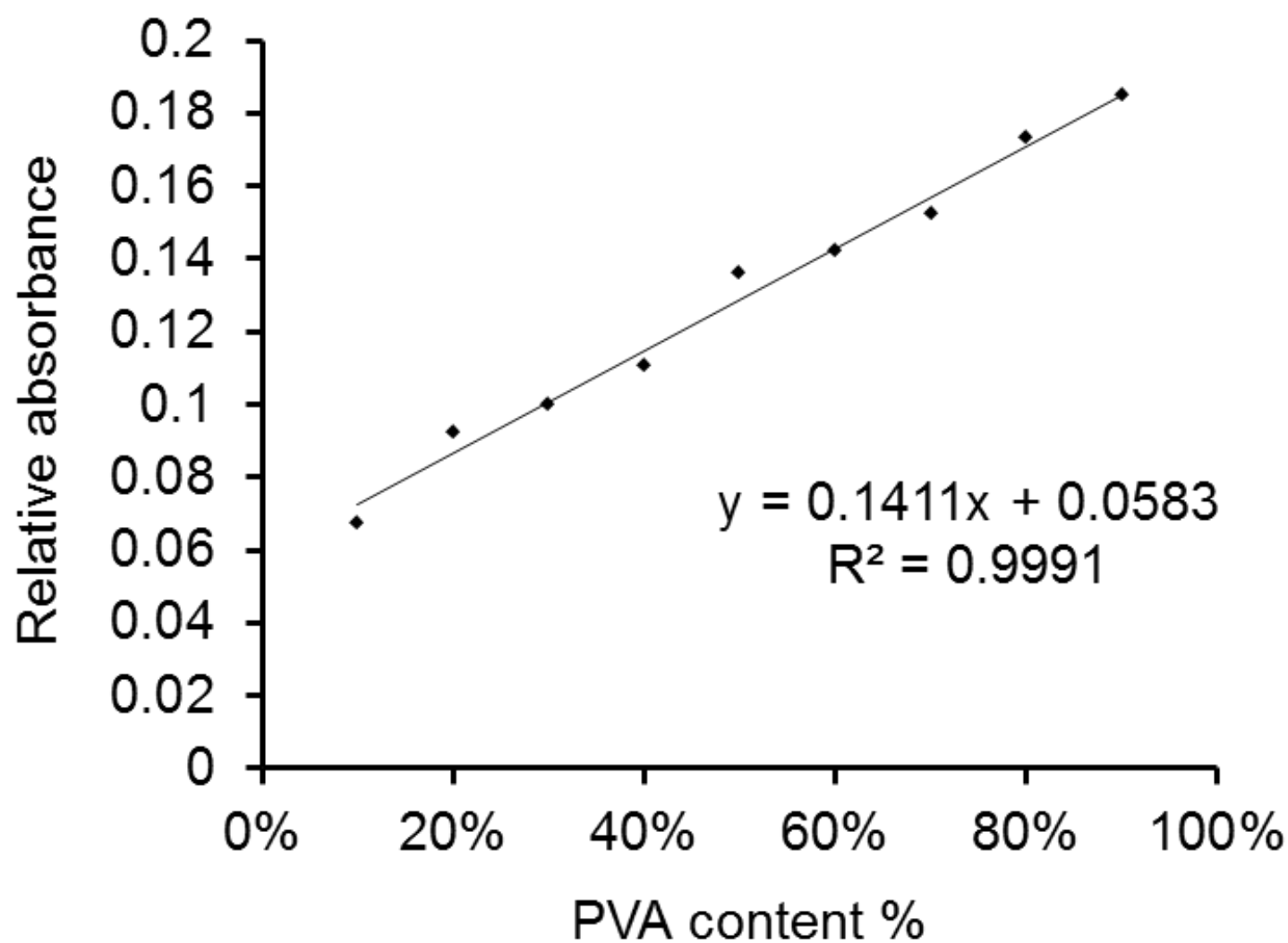

Supplement: Supplementary file 4 — Additional file 4: Fig. S1. The relationship between PVA content and Relative absorbance. [file 12934_2018_909_MOESM4_ESM.pdf]

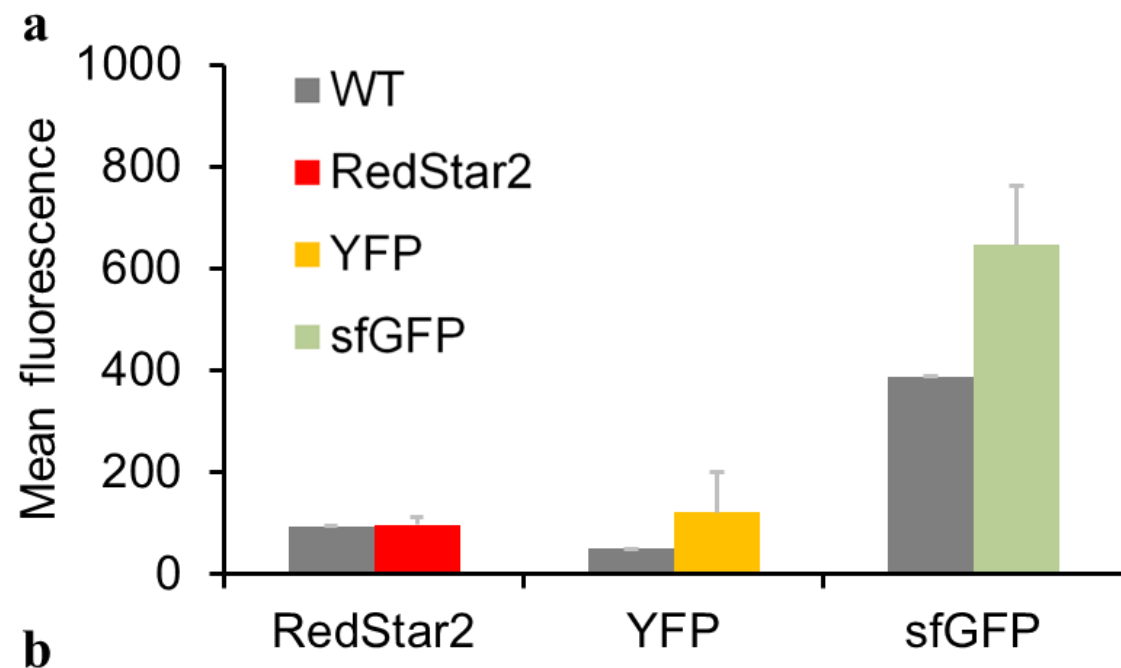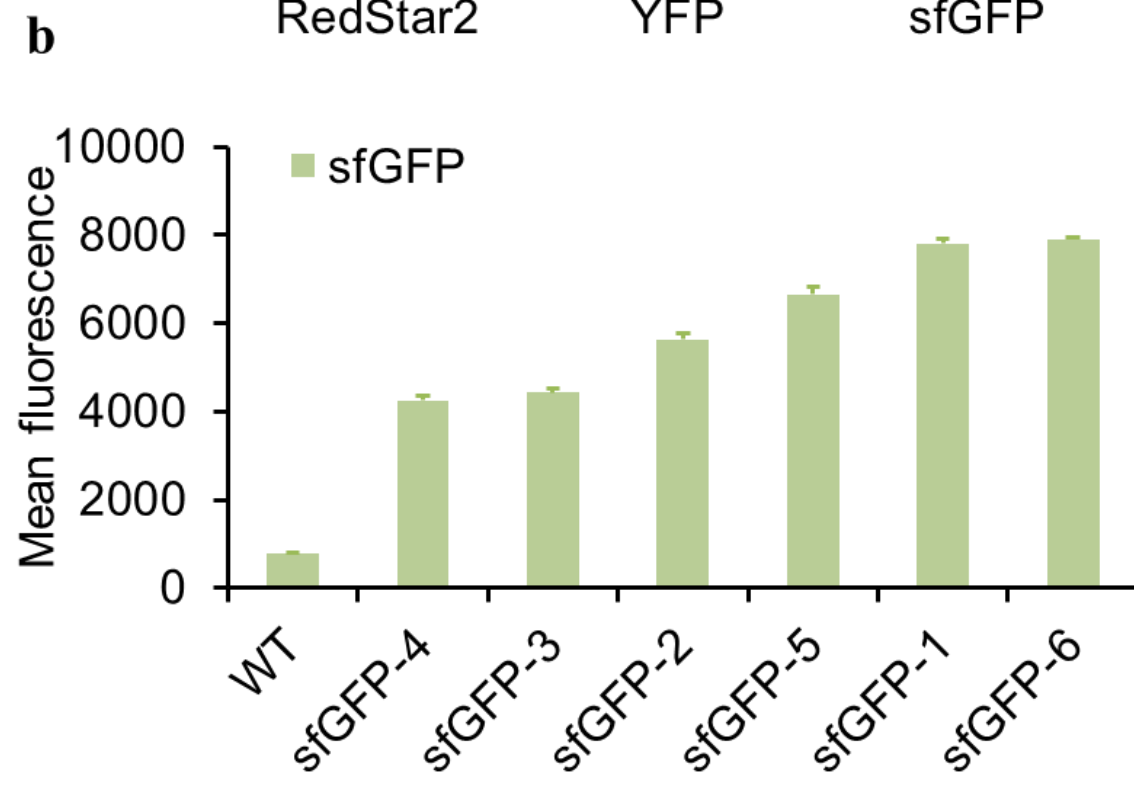

Supplement: Supplementary file 6 — Additional file 6: Fig. S2. GFP-based reporter system in Y. lipolytica. (a) Selection of functional reporter system in Y. lipolytica. The fluorescence levels of RedStar2, YFP and sfGFP driven by the TEFin promoter in Y. lipolytica were tested by multi-mode microplate reader. (b) A synthetic fluorescence-based reporter system containing an sfGFP gene is inserted into the Y. lipolytica genome (the rDNA locus). The mean fluorescence data were collected by multi-mode microplate reader analysis for comparison at a time point of 48 h. The control(con) for these experiments was Y. lipolytica strain without any gene integrated into. The error bars (mean ± SD) were derived from triplicate experiments for each strain. [file 12934_2018_909_MOESM6_ESM.pdf]

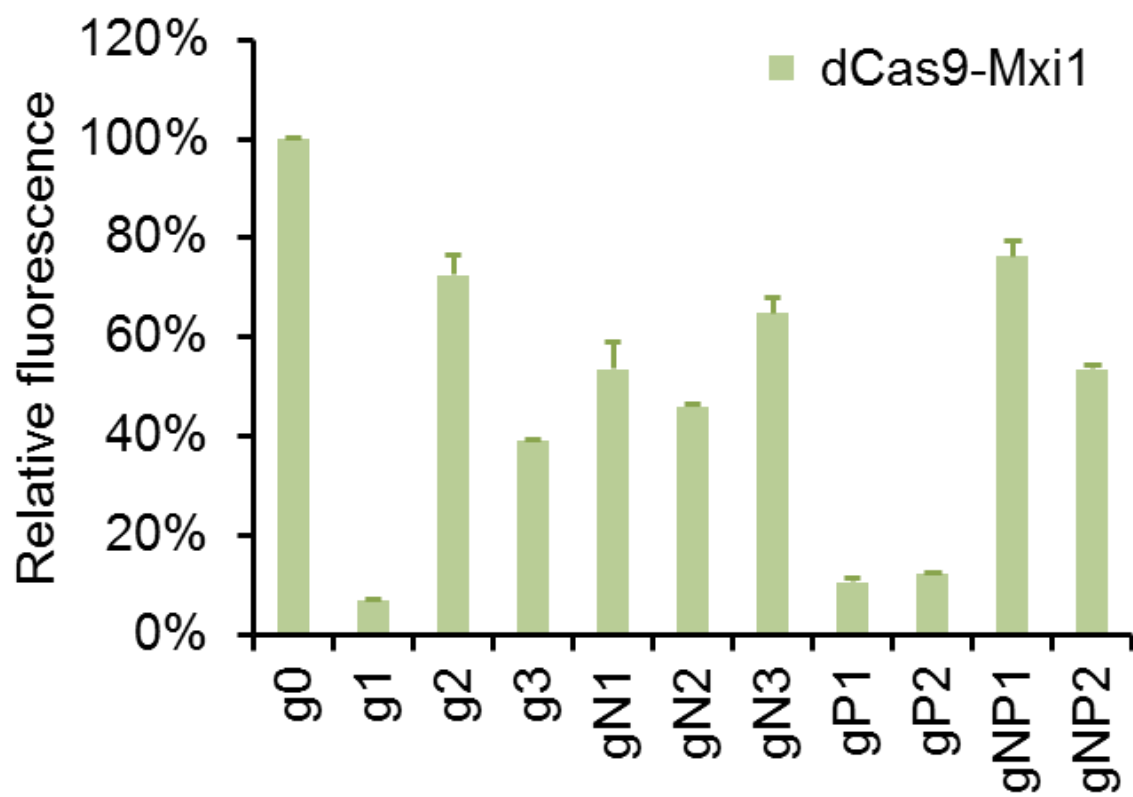

Supplement: Supplementary file 8 — Additional file 8: Fig. S3. Repression of gfp in Y. lipolytica by dCas9-Mxi1. The Mxi1 repressor was fused to the C-terminus of dCas9 and formed the plasmid PMCS-dCas9-Mxi1. CRISPRi repression of gfp with dCas9-Mxi1 complexed with ten gRNAs targeting different regions. The control (g0) shows fluorescence of the cells with dCas9-Mxi1 protein but without the gRNA. The error bars (mean ± SD) were derived from triplicate experiments for each strain. [file 12934_2018_909_MOESM8_ESM.pdf]

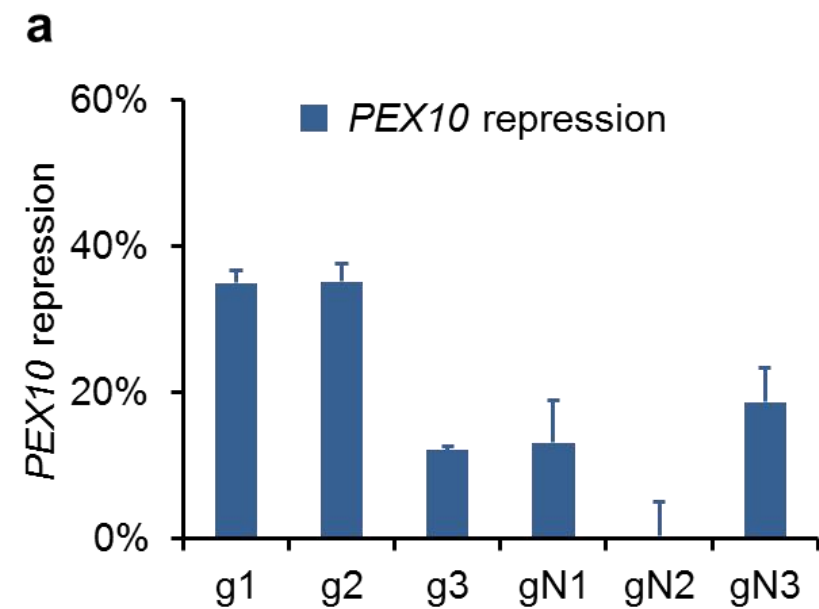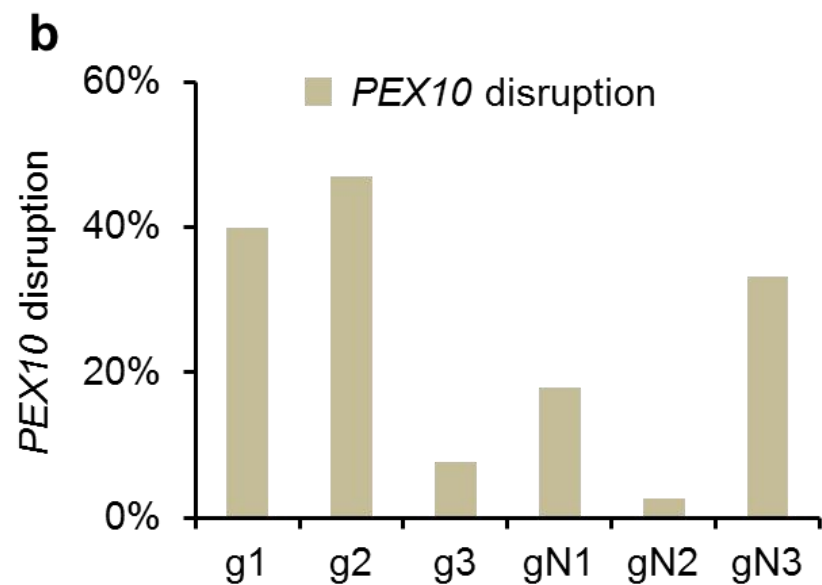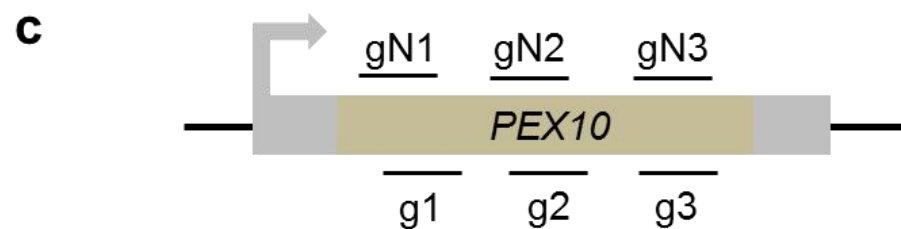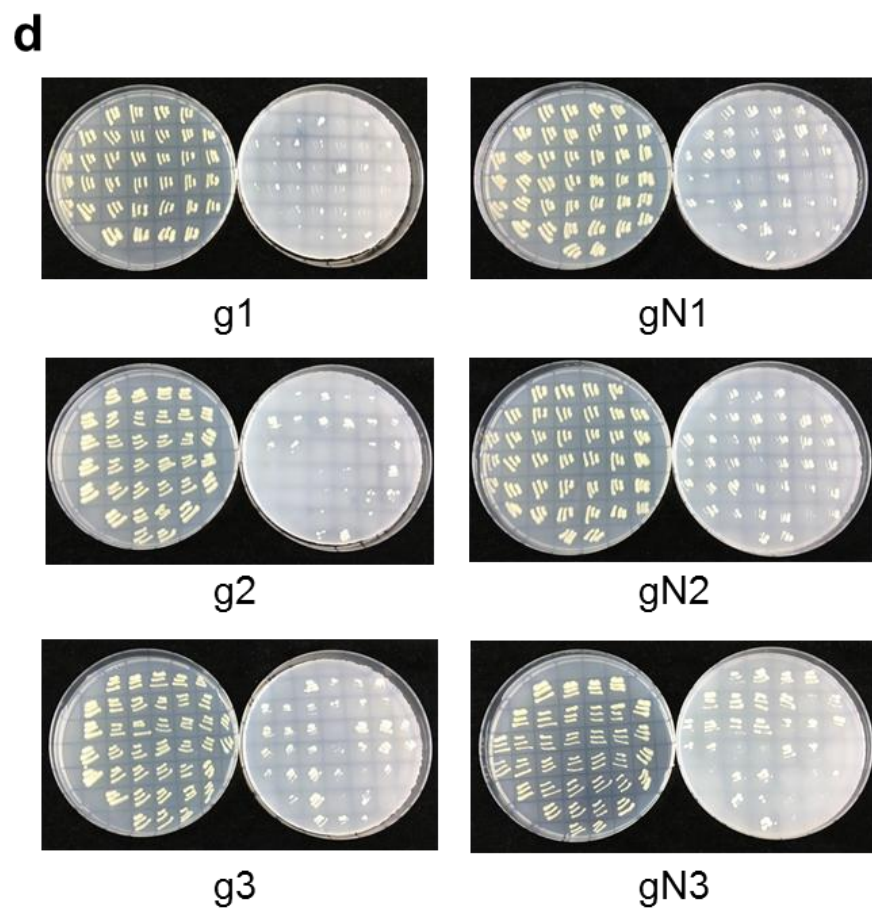

Supplement: Supplementary file 9 — Additional file 9: Fig. S4. Repression and disruption of pex10 in Y. lipolytica by dCas9 and Cas9. (a) Characterization of the pex10 gene’s repression level of six strains interfered by dCas9. (b) Disruption rates of pex10 after 4 days of outgrowth in selective liquid media with different transformed CRISPR-Cas9 plasmids. (c) Placement of gRNA protospacers on the target pex10 gene. (d) Phenotype of pex10 disruptants. Six plates screening of pex10 disrupted phenotypes on SC-Ura and SCO-Ura media. The error bars (mean ± SD) were derived from triplicate experiments for each strain. [file 12934_2018_909_MOESM9_ESM.pdf]

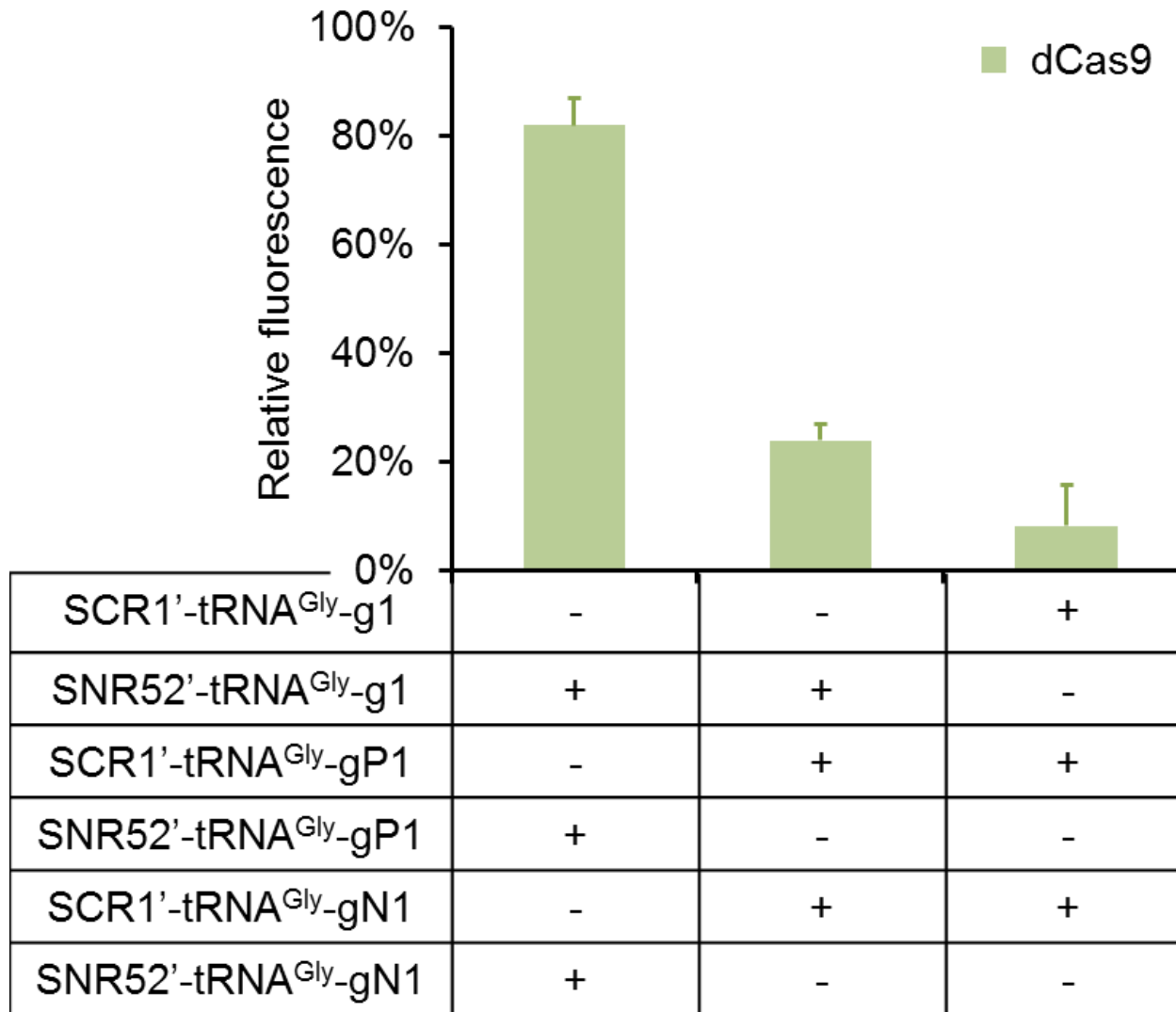

Supplement: Supplementary file 11 — Additional file 11: Fig. S5. Repression of gfp in Y. lipolytica by dCas9-Multi. Efficiency of various gRNA secretion cassettes with different synthetic hybrid promoters on gene repression. The error bars (mean ± SD) were derived from triplicate experiments for each strain. [file 12934_2018_909_MOESM11_ESM.pdf]

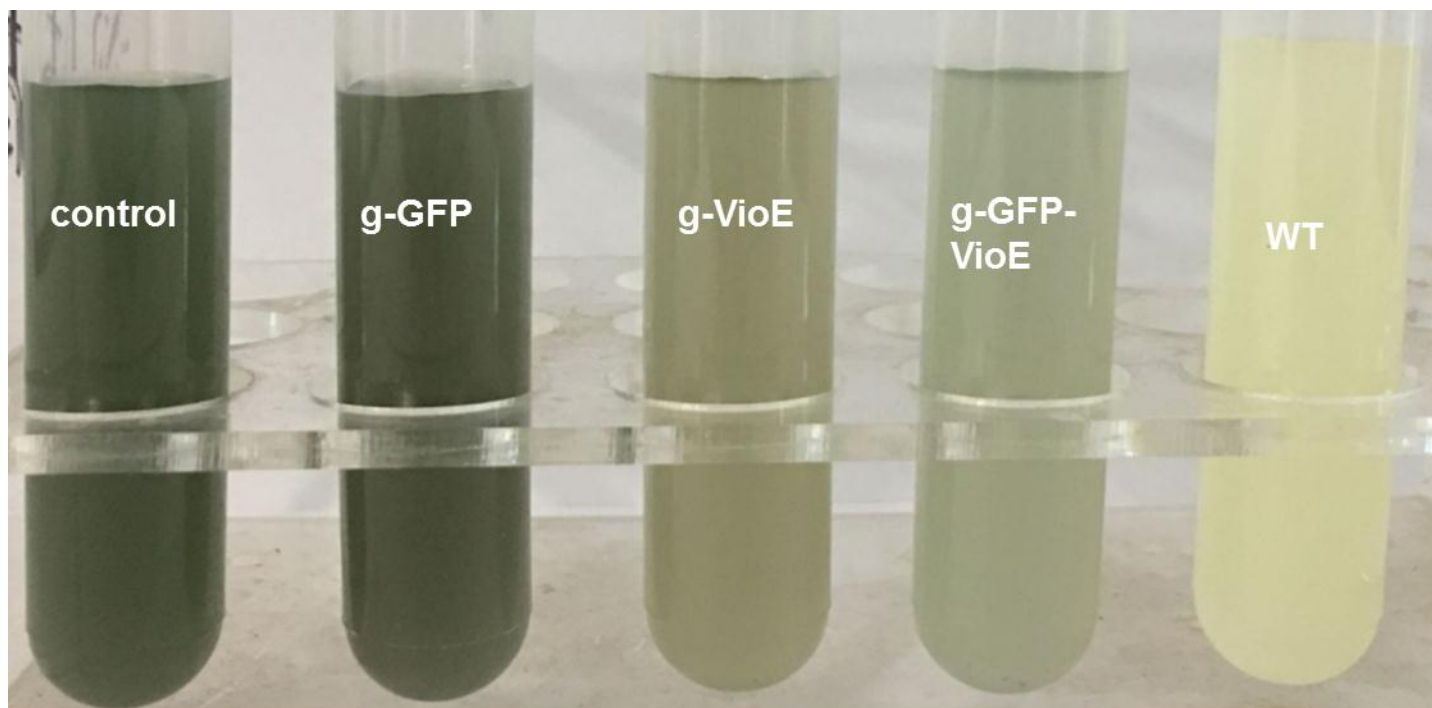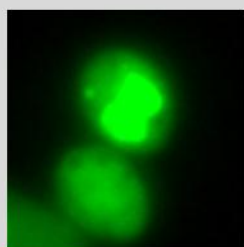

**control**

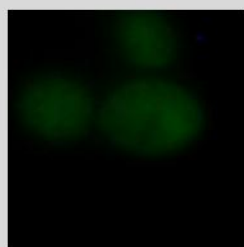

**g-GFP**

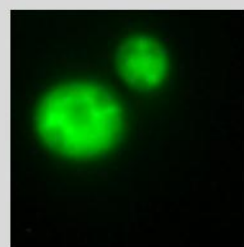

**g-VioE**

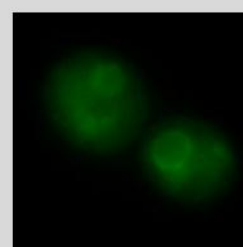

**g-GFP-VioE**

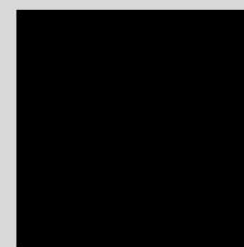

**WT**

Supplement: Supplementary file 12 — Additional file 12: Fig. S6. Photos and its corresponding microscopic images of the interfered strains by dCas9-Multi system. Four related plasmids were transformed into strain VioABE-K8GFP respectively to form four strains: control (means no gRNA towards gfp or vioE, only backbone of plasmid), gRNA-GFP (The dCas9-Multi plasmid only contains gRNA targeting gfp), gRNA-PVA (The dCas9-Multi plasmid only contains gRNA targeting vioE), gRNA-GFP-PVA (The dCas9-Multi plasmid contains gRNAs both targeting gfp and vioE). [file 12934_2018_909_MOESM12_ESM.pdf]
