# Supplementary material for: Gene repression via multiplex gRNA strategy in Y. lipolytica
Source: Microb Cell Fact. 2018 Apr 20;17:62. doi: 10.1186/s12934-018-0909-8 (PMC5910576; doi:10.1186/s12934-018-0909-8)
Supplement: Supplementary file 5 — Additional file 5: Table S3. Primer sequences for RT-PCR. [file 12934_2018_909_MOESM5_ESM.docx]

**Table S3. Primer sequences for RT-PCR**

| Name | Sequence |
| --- | --- |
| Yarrowia-act-F | CTCCATCAAGGTCAAGAT |
| Yarrowia-act-R | TACCAAGAGAAGCAAGAA |
| GFP-FP-F | ACTTCAAGGAGGACGGTAAC |
| GFP-FP-R | TTGTCGGCGGTGATGTAG |
