# Supplementary material for: Gene repression via multiplex gRNA strategy in Y. lipolytica
Source: Microb Cell Fact. 2018 Apr 20;17:62. doi: 10.1186/s12934-018-0909-8 (PMC5910576; doi:10.1186/s12934-018-0909-8)
Supplement: Supplementary file 7 — Additional file 7: Data S2. The details for constructing strain VioABE, YL-GFP and VioABE-K8GFP. [file 12934_2018_909_MOESM7_ESM.docx]

**Additional file 7:** Data S2

The details for constructing strain VioABE, YL-GFP and VioABE-K8GFP

1, VioABE: The genes including *vioA,vioB,vioE* were synthesized by GenScript, after digested by BsaI, BsmBI, BsaI separately, the corresponding segments were ligated into three different cassettes to get sequential expression modules. The three different cassettes were on plasmid pLD01 owning standard left 5’-CATT-3’ and right 5’-TAAA-3’ sticky ends for ligation with *vioA* gene, plasmid pLD02in owning standard left 5’-CTGC-3’ and right 5’-TAAA-3’ sticky ends for ligation with *vioB* gene, and plasmid pLD03 owning standard left 5’-CATT-3’ and right 5’-TAAA-3’ sticky ends for ligation with *vioE* gene, respectively (plasmids pLD01, pLD02in and pLD03 were constructed by overlap and ligated with pEASY-Blunt backbone). The constructed expression cassettes were: H0-EXP1p-VioA-xpr2t-H1, H1-TEF1p-VioB-lip2t-H2, H2-GPDp-VioE-OCTt –H3. All “Hx” were homologous arms at the length of 200 bp with low G/C contents (20%-30%). Then the three cassettes were digested by NotI and the target fragments were recovered by gel extraction kit. Then an equimolar mixture of each segment with an extra left module of “rDNAL-Hph-H0” and a right module of “H3-rDNAR” were mixed to form a total 25 μl for further transformation into the *Y. lipolytica* and the strain VioABE was constructed (The “rDNAL” was 700 bp upstream of rDNA’s ORF, and “rDNAR” was 700 bp downstream of rDNA’s ORF).

2, YL-GFP: The construction method of strain YL-GFP was similar with VioABE. The synthesized gene sfGFP was digested by BsaI and the segment was ligated into pLD01 cassette, forming H0-EXP1p-sfGFP-xpr2t-H1 expression cassette. Then the constructed cassette was digested by NotI and the target fragments was recovered by gel extraction kit. Then an equimolar mixture of H0-EXP1p-sfGFP-xpr2t-H1 and H1-H2 expression cassette together with an extra left module of “rDNA-Hph-H0” and a right module of “H2-rDNAR” were mixed to form a total 25 μl for further transformation into the *Y. lipolytica* and the strain YL-GFP was constructed.

3, VioABE-K8GFP: The VioABE was used as the backbone strain for constructing strain VioABE-K8GFP. The synthesized gene *sfGFP* was ampliﬁed with the primers BsaI-K8-GFP-F and BsaI-K8-GFP-R. Plasmid K8F8 owns standard left 5’-CTGG-3’ and right 5’-TAAA-3’ sticky ends. Then the fragment was digested by BsaI and the segment was ligated into K8F8 cassettes, forming ku80L-FBAinp-sfGFP-OCTt- ku80R expression cassette. The constructed cassette was digested by NotI and the target fragments was recovered by gel extraction kit. Then 5ul of ku80L-FBAinp-sfGFP-OCTt- ku80R was transformed into the VioABE backbone strain. The transformed cells were plated on SC-Ura-Hph plates. (The “ku80L” was 1000 bp upstream of ku80’s ORF, and “ku80R” was 1000 bp downstream of ku80’s ORF)
