# Supplementary material for: Gene repression via multiplex gRNA strategy in Y. lipolytica
Source: Microb Cell Fact. 2018 Apr 20;17:62. doi: 10.1186/s12934-018-0909-8 (PMC5910576; doi:10.1186/s12934-018-0909-8)
Supplement: Supplementary file 10 — Additional file 10: Data S3. The details for constructing Cas9-pex10 plasmids and transformation method. [file 12934_2018_909_MOESM10_ESM.docx]

**Additional file 10:** Data S3

The details for constructing Cas9-pex10 plasmids and transformation method

1, Plasmids construction: The backbone plasmid used for constructing plasmids for gene knockout n in *Y. lipolytica* was pMCS-Cen1. The synthesized gRNA was incorporated into pMCS-URA *via* one-step Golden Gate assembly. The corresponding plasmids were digested with restriction enzyme BamHI and HindIII, and the segment of *Cas9* was ligated into to form the final plasmids.

2, Transformation method: Frozen-EZ kit was used for *Y. lipolytica* transformations. The difference from other transformation was that after adding 500ul EZ3, 200ul of the mixture was introduced to 25 ml polypropylene tube containing 5 ml fresh Sc-Ura medium and cultured for 4 days, then diluted and coated SC-URA plate, other than coated plates directly. The correct tranfomants of *Y. lipolytica* were selected and screened for on SC-Ura and SCO-Ura (0.67% yeast nitrogen base, 0.2% amino acid mixture, 0.3% Oleic acid and 0.4% polysorbate 40).
